# Cell-free expression of RNA encoded genes using MS2 replicase

**DOI:** 10.1093/nar/gkz817

**Published:** 2019-09-30

**Authors:** Laura I Weise, Michael Heymann, Viktoria Mayr, Hannes Mutschler

**Affiliations:** 1 Biomimetic Systems, Max Planck Institute of Biochemistry, Martinsried 82152, Germany; 2 Dept. Cellular and Molecular Biophysics, Max Planck Institute of Biochemistry, Martinsried 82152, Germany

## Abstract

RNA replicases catalyse transcription and replication of viral RNA genomes. Of particular interest for *in vitro* studies are phage replicases due to their small number of host factors required for activity and their ability to initiate replication in the absence of any primers. However, the requirements for template recognition by most phage replicases are still only poorly understood. Here, we show that the active replicase of the archetypical RNA phage MS2 can be produced in a recombinant cell-free expression system. We find that the 3′ terminal fusion of antisense RNAs with a domain derived from the reverse complement of the wild type MS2 genome generates efficient templates for transcription by the MS2 replicase. The new system enables DNA-independent gene expression both in batch reactions and in microcompartments. Finally, we demonstrate that MS2-based RNA-dependent transcription-translation reactions can be used to control DNA-dependent gene expression by encoding a viral DNA-dependent RNA polymerase on a MS2 RNA template. Our study sheds light on the template requirements of the MS2 replicase and paves the way for new *in vitro* applications including the design of genetic circuits combining both DNA- and RNA-encoded systems.

## INTRODUCTION

**Figure 1.**
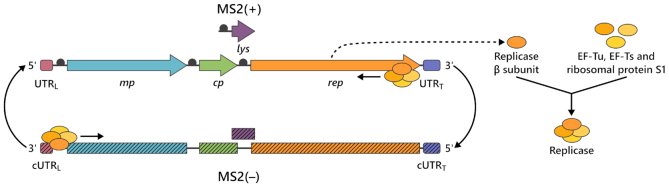
Organization and replication of the 3569 nt ssRNA MS2 genome. The genome encodes for four genes. The maturation protein (*mp*) for cell entry, the coat protein (*cp*) forming the capsid, the lysis protein (*lys*) for host lysis and the replicase β subunit (*rep*). The coding part of the genome is flanked by two untranslated regions (UTRs): The 5′ UTR leader sequence (UTR_L_), and the 3′ UTR trailer sequence (UTR_T_). Replicase β subunit associates with the host factors EF-Tu, EF-Ts, and the ribosomal protein S1 to form the functional MS2 replicase. The replicase initiates (−) strand synthesis from the UTR_T_ of the (+) strand and (+) strand synthesis from the complementary UTR_L_ (cUTR_L_) of the (−) strand. Black dots are ribosome binding sites (RBS). Arrows on the (+) strand indicate open reading frames and boxes UTR elements. Their respective reverse complements on the (−) strand are indicated as boxes with dashed fillings.

The RNA coliphage MS2 is one of the oldest model systems of modern molecular biology and its detailed investigation has led to numerous fundamental findings and applications. For example, its genome was the first to be completely sequenced, revealing for the first time the genetic organization of a biological entity (1). The small (+) strand 3569 nucleotide (nt) single-stranded RNA (ssRNA) genome encodes for only four proteins: a maturation protein required for adhesion and cell entry into its bacterial hosts, a coat protein for capsid formation and RNA packaging, a lysis gene required for virion release at the end of the infection cycle, and the catalytic replicase β subunit (rep β subunit) required for RNA replication by the replicase heterocomplex (Figure 1). Further studies led to the discovery of RNA–RNA and RNA–protein interactions that control the precise timing and strength of viral protein expression during the bacteriophage life cycle (2–4). From these interactions, binding of the coat protein to a ‘translational operator’ stem–loop containing the start codon of the rep β subunit (4) has become a versatile tool in molecular and cell biology applications such as RNA imaging (5–7).

Primer-independent replication of MS2 (+) strand genomes proceeds via complementary (−) strand intermediates, which serve as templates for efficient transcription of viral progeny (+) strands (Figure 1) (8,9). The replicase complex responsible for this process was among the first active viral RNA-dependent RNA polymerases that could be purified and studied in isolation (10,11). However, its limited stability and difficult purification protocol at that time (9,10) prevented a detailed characterization such as the molecular principles that confer specificity during MS2 genome replication. Instead, most of our knowledge about phage RNA replication comes from studies on the replicase from the closely related bacteriophage Qβ. The Qβ replicase (and presumably also the MS2 replicase) holoenzyme (hereafter referred to only as replicase) form through association of the catalytic rep β subunit with three bacterial host factors: the ribosomal protein S1 (α subunit), the elongation factors EF-Tu (γ subunit), and EF-Ts (δ subunit) (9,12–15). However, even in the Qβ replicase, the exact roles of all host factors are still under debate. Both γ and δ subunit appear to act as chaperones for the rep β subunit (16) and are essential for processive RNA elongation (17,18), while the α subunit seems to be necessary for RNA initiation and termination (19).

The Qβ replicase was central in a series of pioneering molecular evolution experiments that led to the discovery of a small ‘RQ’ RNAs (20) including the famous ‘Spiegelman's monster’ (21), which are efficiently replicated in the presence of the Qβ replicase. Some RQ RNAs can even be used as scaffolds for the amplification and evolution of mRNAs in cell-free expression systems (22–27). However, one of the disadvantages of using such small ‘selfish’ RNA replicators as scaffolds for *in vitro* gene expression is the general difficulty of designing RNA constructs that are still suitable as efficient replication templates (20,25,27–29). Tedious steps of incremental RNA secondary structure optimization are often needed (27,30,31) to prevent formation of longer RNA duplexes unfit for replication and translation (32,33). Moreover, existing scaffolds are not suited to decouple transcription and RNA replication, which complicates experiments in which, for example, RNA amplification is not desired.

In this study we show that the active MS2 replicase can be synthesized in a recombinant, *Escherichia coli*-based *in vitro* transcription-translation system (PURE – Protein synthesis Using Recombinant Elements (34)). We find that the *de novo* synthesized replicase can catalyse transcription of mRNAs using engineered antisense RNA constructs as templates. This DNA-free *in vitro* transcription activity enables RNA-dependent transcription-translation of non-viral genes in both batch reactions and reactions encapsulated in water-in-oil emulsion droplets. Finally, we demonstrate that the new MS2-based system can be used to link *in vitro* DNA- and RNA-dependent transcription-translation reactions enabling informational coupling between otherwise orthogonal genetic systems. Thus, in addition to shedding light on the template requirements of the poorly characterized MS2 replicase, the new cell-free RNA expression system could enable novel directed evolution strategies or the design of genetic circuits involving RNA in a secondary genome.

## MATERIALS AND METHODS

### Preparation of DNA/RNA constructs

The preparation of all DNA and RNA constructs is described in the Supplementary Methods. Primers used in this study are listed in Supplementary Table S1, ribosome binding sites (RBS) are listed in Supplementary Table S2, and the final sequences of all gene constructs are listed in Supplementary Table S3.

### *In vitro* protein synthesis

*In vitro* protein synthesis was performed using the PURExpress^®^ system (NEB), which utilizes two solutions: solution A (tRNAs, rNTPs, amino acids and other small molecules) and solution B (ribosomes and proteins including T7 DNA-dependent RNA polymerase) (35). The total volume for a standard PURE reaction was 12.5 μl, consisting of 5 μl Solution A, 3.75 μl Solution B and other added components as indicated in the corresponding sections. All reactions were set up on ice. If necessary, the final reaction volume was adjusted with nuclease-free water (Thermo Fisher Scientific).

### Fluorescent labelling and imaging of *in vitro* expressed MS2 replicase β subunit

A standard PURE reaction programmed with 17.5 nM (250 ng) of a linear Rep DNA template (under control of a T7 promoter) was supplemented with 0.6 µl FluoroTect™ Green_Lys_ tRNA (FluoroTect™ Green_Lys_*in vitro* translation labelling system, Promega). Template DNA was omitted in the negative control reaction. Samples were incubated for 2  h at 37 °C in a nuclease-free PCR tube (Thermo Fisher Scientific) using a ProFlex PCR System (Thermo Fisher Scientific) and subsequently treated with 0.6 μl RNase Cocktail™ Enzyme Mix (0.5 U/μl RNase A and 20 U/μl RNase T1, Thermo Fisher Scientific) for 15 min at 37°C to degrade non-incorporated Green_Lys_ tRNA. 7.5 μl sample were mixed with an equal volume 2× Laemmli sample loading buffer (incl. 200 mM DTT) and denatured for 2.5 min at 65°C. Samples were analysed by conventional discontinuous SDS-PAGE (10% gel) run at 4°C (100 V for 10 min, then 200 V) on a Midi-format electrophoresis system (Atto). The fluorescent signal of the *de novo* expressed rep β subunit was imaged on a fluorescence laser scanner (Typhoon FLA 9000, GE Healthcare) at either 473 nm (blue LD laser/510LP filter) or at 532 nm (green SHG laser/575LP filter). Total protein and the molecular-weight size marker (PageRuler™ Unstained Protein Ladder, Thermo Fisher Scientific) were visualized after SYPRO Ruby (Bio-Rad) staining using the same instrument (473 nm, blue LD laser/575LP filter).

### Real-time fluorescence measurements of MS2 RdTT

Design and synthesis of individual readout constructs (Supplementary Table S3) are described in detail in the Supplementary Methods. Typical RNA constructs consist of antisense strands embedded between the MS2 cUTRs ([*gene*]_MS2_ (−) RNA). For real-time detection, standard PURE reactions were supplemented with the following final concentrations of DNA or RNA templates and reagents/additives:

F30-Broccoli transcription by MS2 replicase: 70 nM MS2-rep/MS2-ddrep (+) RNA, 350 nM [F30-Bro]_MS2_ (−) RNA, 6% (w/v) PEG 4000 and 10 μM DFHBI-1T fluorophore. *β-gal α-complementation by MS2 RdTT*: 70 nM MS2-rep/MS2-ddrep (+) RNA, 70 nM [*α*]_MS2_ (−) RNA, 70 nM ω-protein and 50 μM FDG substrate. *sfGFP expression by MS2 RdTT*: 70 nM MS2-rep/MS2-ddrep (+) RNA, 70 nM [*sfGFP*]_MS2_ (−) RNA and 6% (w/v) PEG 4000. *Full-length β-gal expression by MS2 RdTT*: 70 nM MS2-rep/MS2-ddrep (+) RNA, 70 nM [*lacZ*]_MS2_ (−) RNA, 6% (w/v) PEG and 50 μM FDG substrate. *Comparison +/– cUTR_T_ for F30-Broccoli transcription by MS2 replicase*: 70 nM MS2-rep/MS2-ddrep (+) RNA, 350 nM standard [F30-Bro]_MS2_ (−) RNA/truncated 350 nM [F30-Bro]_MS2_ (−) RNA, 6% (w/v) PEG 4000 and 10 μM DFHBI-1T fluorophore*. Comparison +/– cUTR_T,_ for sfGFP expression by MS2 RdTT*: 70 nM MS2-rep/MS2-ddrep (+) RNA, 70 nM standard [*sfGFP*-RBS_2_]_MS2_ (−) RNA/70 nM truncated [*sfGFP*-RBS_2_]_MS2_ (−) RNA and 6% (w/v) PEG 4000. *SP6 pol expression by MS2 RdTT coupled with sfGFP expression by SP6 DdTT*: 70 nM MS2-rep/MS2-ddrep (+) RNA, 35 nM [*SP6 pol*]_MS2_ (−) RNA, 16 nM (125 ng) SP6-sfGFP DNA, 5,5% (w/v) PEG 4000 and ∼1 U RNase inhibitor (moloX) per μl reaction. Note that the latter reaction was slightly diluted (13.5 μl versus 12.5 μl) due to the high amount of additives required to perform the reaction.

All reactions were prepared in MicroAmp Fast 8-Tube Strips (Thermo Fisher Scientific) and incubated at 37°C in a StepOne Real-Time PCR System (Thermo Fisher Scientific). Fluorescence signals were recorded every 60 s (total incubation time was 4 h). If not stated otherwise, all experiments were performed in technical triplicates from single master mixes.

### In-gel imaging and quantitation of *in vitro* transcribed [F30-Bro]_MS2_ (+) RNA

An appropriately up-scaled standard PURE reaction (∼10-fold) was programmed with 70 nM MS2-rep (+) RNA, 70 nM [F30-Bro]_MS2_ (−) RNA, 6% (w/v) PEG 4000 and ∼1 U RNase inhibitor (NEB) per μl reaction. Reactions were incubated at 37 °C in a nuclease-free PCR tube (Thermo Fisher Scientific) using a ProFlex PCR System (Thermo Fisher Scientific). Sample aliquots (5 μl) were taken at different time points (0, 15, 30, 60, 90 and 120 min), mixed with 5× native RNA loading buffer (50 mM Tris–HCl pH 8, 100 mM EDTA pH 8, 25% (v/v) glycerol, 0.05% (w/v) bromophenol blue), shock-frozen in liquid nitrogen and stored at −80°C until further use. To create standard curves for in-gel F30-Broccoli fluorescence detection, *in vitro* transcribed [F30-Bro]_MS2_ (+) RNA (Supplementary Methods) was diluted in nuclease-free H_2_O (supplemented with 0.02% (w/v) PEG 4000 to prevent RNA adhesion to the tube surface at low concentrations) (36) to final concentrations of 15, 30, 45, 75 and 120 nM. Aliquots of the diluted standards were shock-frozen in liquid nitrogen and stored at −80°C until further use. Due to slight but significant deviations of the integrated in-gel fluorescence from linearity at low F30-Broccoli concentrations, the standard curve was empirically fitted with a square polynomial (see Supplementary Files). Furthermore, in-gel F30-Broccoli fluorescence in samples containing PURE components was quenched by about 23% compared to [F30-Bro]_MS2_ (+) RNA standards that were diluted in H_2_O. Thus, to enable a more precise quantification of the *de novo* transcribed [F30-Bro]_MS2_ (+) RNA concentrations in MS2 transcription reactions, all fluorescence values were therefore multiplied with a correction factor of 1.3 (see Supplementary Files), which was determined by quantifying and comparing equivalent amounts of [F30-Bro]_MS2_ (+) RNA diluted either in H_2_O or PURE reaction buffer.

All samples and the standards were analysed by native TBE-PAGE (5% Mini-Protean^®^ TBE Gel, Bio-Rad) run at 100 V in 1× TBE on a Mini-format electrophoresis system (Bio-Rad). RiboRuler Low Range RNA Ladder (Thermo Fisher Scientific) was used as molecular weight standard. Following electrophoresis, gels were washed 3 × 5 min with ultrapure H_2_O and then stained for 20 min in 20 ml DFHBI-1T staining solution (10 μM DFHBI-1T, 40 mM HEPES pH 7.4, 100 mM KCl, 1 mM MgCl_2_). The fluorescent signal of the *de novo* transcribed [F30-Bro]_MS2_ (+) RNA was imaged on a fluorescence laser scanner (Typhoon FLA 9000, GE Healthcare) at 473 nm (blue LD laser/510LP filter). Gel band intensities were quantified using ImageQuant TL 1D 7.0 (GE Healthcare). Brightness and contrast adjustments of the displayed gel image were applied homogenously.

### Microfluidic device fabrication, droplet generation and microscopy

Droplet nozzles were fabricated through soft lithography with final chips cast from SU8 masters as 10:1 base:curing agent poly-(dimethylsiloxane) (PDMS, Sylgard), bonded to glass using oxygen plasma and surface treated with cytop 809M (AGC Chemicals Europe) to be fluorophilic, as previously described (37). Monodisperse droplets with a diameter of about 50 μm were produced at room temperature by flow focusing the aqueous phase with a fluorinated oil phase (Novec 7500, 3M) and stabilized against coalescence with a biocompatible surfactant (Pico-Surf 1, 2% (w/w) in Novec 7500; Sphere Fluidics Limited). Before encapsulation in water-in-oil emulsion droplets, PURE reactions were prepared in PCR tubes and stored on ice. F30-Broccoli transcription reactions and β-gal expression reactions by MS2 RdTT (Supplementary Figure S6) were prepared the same way as the batch reactions described above with the only difference being, for the latter, the antisense RNA template was replaced by [*lacZ*-RBS_2_]_MS2_ (−) RNA (containing a weaker ribosome binding site) at a concentration of 60 nM. For compartmentalization, samples were aspirated from the PCR tubes into the PTFE tubing by operating syringe pumps (neMESYS, Cetoni) in withdrawal mode at 4000 μl/h. Subsequently, the filled sample tubes were connected to the inlets of the PDMS chip and injected at a flow-rate of 400 μl/h for the aqueous phase and the oil phase each. The resulting emulsion droplet creams were loaded into rectangular 50 × 500 μm glass capillaries (VitroTubes) by capillary action. For imaging, the filled glass capillaries were placed on a glass microscope slip and capillary inlets were sealed with 5 Minute-Epoxy to prevent evaporation. All images were taken on a LSM 780 confocal laser scanning microscope equipped with a custom environmental chamber pre-heated to 37°C and a Plan-Apochromat 10×/0.45 M27 objective (Carl Zeiss). Green fluorescence of the droplets was excited using the 488 nm Argon laser on the first channel (Fluorescence) with the corresponding filter (λ_em_ = 559 nm), while transmission bright-field images were also collected to access overall droplet shapes throughout the experiment. Time series for both reactions were acquired with 3 min imaging intervals for 80 cycles. The images in Figure 6, Supplementary Figure S6 and the Supplementary Movies show a merge of both fluorescence and bright-field channels. The microscope images and the Supplementary Movies were processed with ZEN (Carl Zeiss) and analysed using Fiji v1.52j. To assess the fluorescence change of individual droplets during the experiment, the mean fluorescence of eight random droplets was plotted over time. Brightness and contrast adjustments of the displayed images or movies were applied homogenously.

## RESULTS

### *In vitro* synthesized MS2 replicase enables general MS2 RdTT of non-viral genes

**Figure 2.**
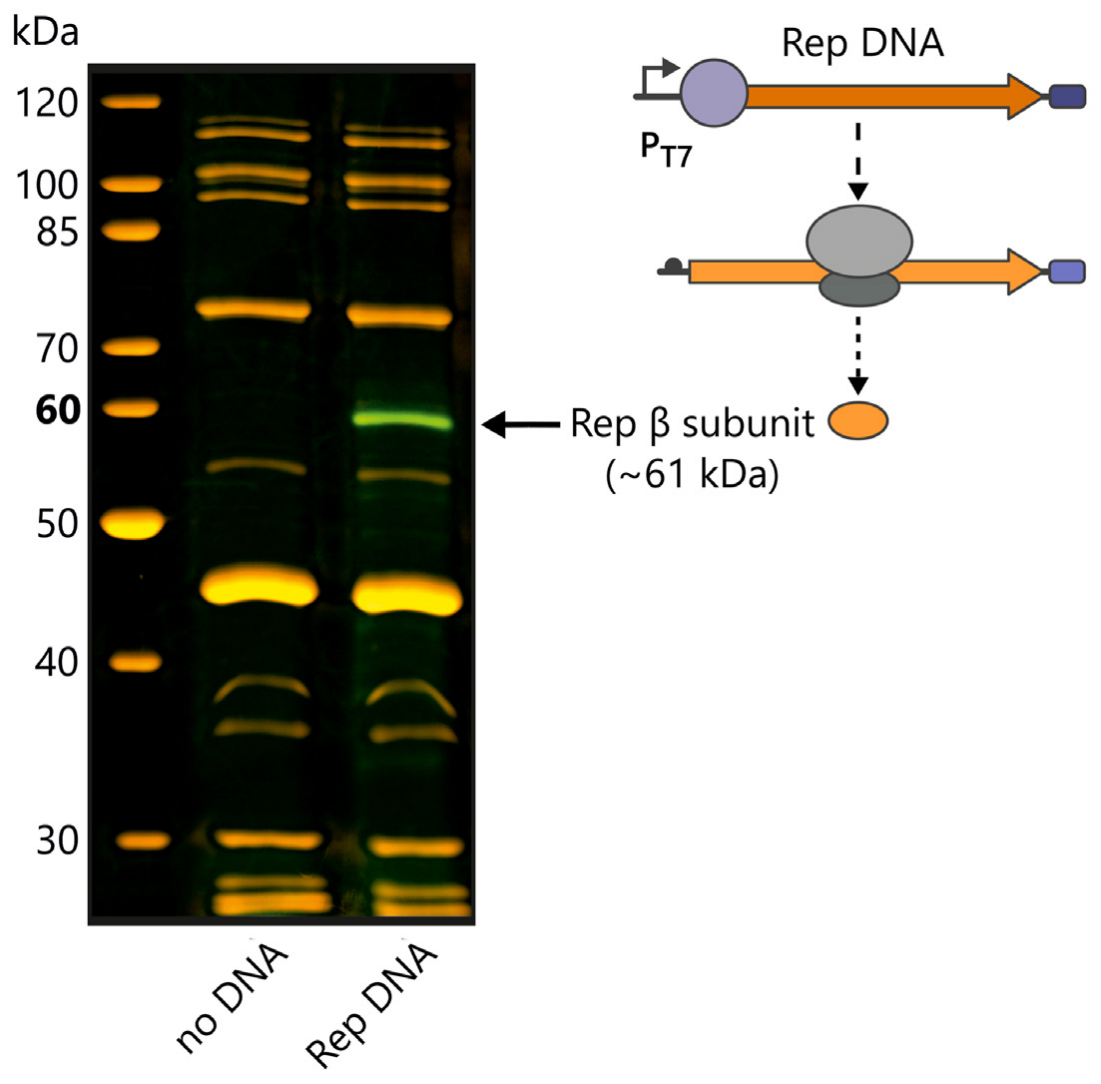
MS2 replicase β subunit expression in the PURE system. *De novo* synthesized rep β subunit (green) can be visualized after Green_Lys_ labelling by SDS-PAGE. The PURE reaction was programmed with 17.5 nM of a linear DNA template encoding the rep β subunit (Rep DNA) under control of a T7 promoter (P_T7_) enabling T7 DNA-dependent transcription-translation (T7 DdTT) (right panel). SYPRO Ruby staining (orange) was used to visualize all other proteins present in the PURE system. No fluorescent protein band is visible in the absence of a DNA template (no DNA).

The MS2 replicase forms *in vivo* presumably from the association of the catalytic rep β subunit with *E. coli* host factors similar to the Qβ replicase (9,12–15) (Figure 1). To probe whether active MS2 replicase can also be produced *in vitro*, we first tested conventional DNA-dependent transcription-translation (DdTT) of the rep β subunit in a commercial PURE system, which contains the T7 DNA-dependent RNA polymerase (T7 pol). To this end, we engineered a linear DNA construct encoding a minimized version of the MS2 genome devoid of all coding sequences except for the rep β subunit with an upstream T7 promotor (Rep DNA). Using this construct, we could indeed detect *de novo* synthesis of the 60.8 kD rep β subunit in the PURE system by the Green_Lys_ labelling approach and conventional SYPRO Ruby staining in yields matching the concentration of the translation factors present in the PURE system (Figure 2).

Next, we set out to probe if the expressed rep β subunit can form an active replicase through complex formation with the proposed *E. coli* host factors, which are present in the PURE system (34). RNA replication during the life cycle of (+) ssRNA viruses is typically highly asymmetric with the genomic (−) ssRNA being the better transcription template (38) to prevent formation of biologically inert RNA duplexes (39,40). Therefore, we sought to detect replicase activity by the conversion of (−) ssRNA to (+) ssRNA, i.e. RNA-dependent RNA transcription. We anticipated that template recognition by the replicase relies on specific 5′ and 3′ terminal RNA secondary structure elements, similar to other RNA viruses (41–44). In particular, we expected that the responsible RNA domains are contained in the two untranslated regions (UTRs) of the MS2 genome, each of which folds into a defined secondary structure (45). The UTR leader sequence (129 nt, UTR_L_) is located at the 5′ end of the (+) strand genome and the UTR trailer sequence (181 nt, UTR_T_) overlaps with the end of the rep β subunit gene at the 3′ end (Figure 1). We thus wanted to verify if the reverse complements of the UTR_L_ (cUTR_L_) and the UTR_T_ (cUTR_T_) found in the genomic (−) strand are sufficient for general template recognition and transcription initiation by the MS2 replicase.

**Figure 3.**
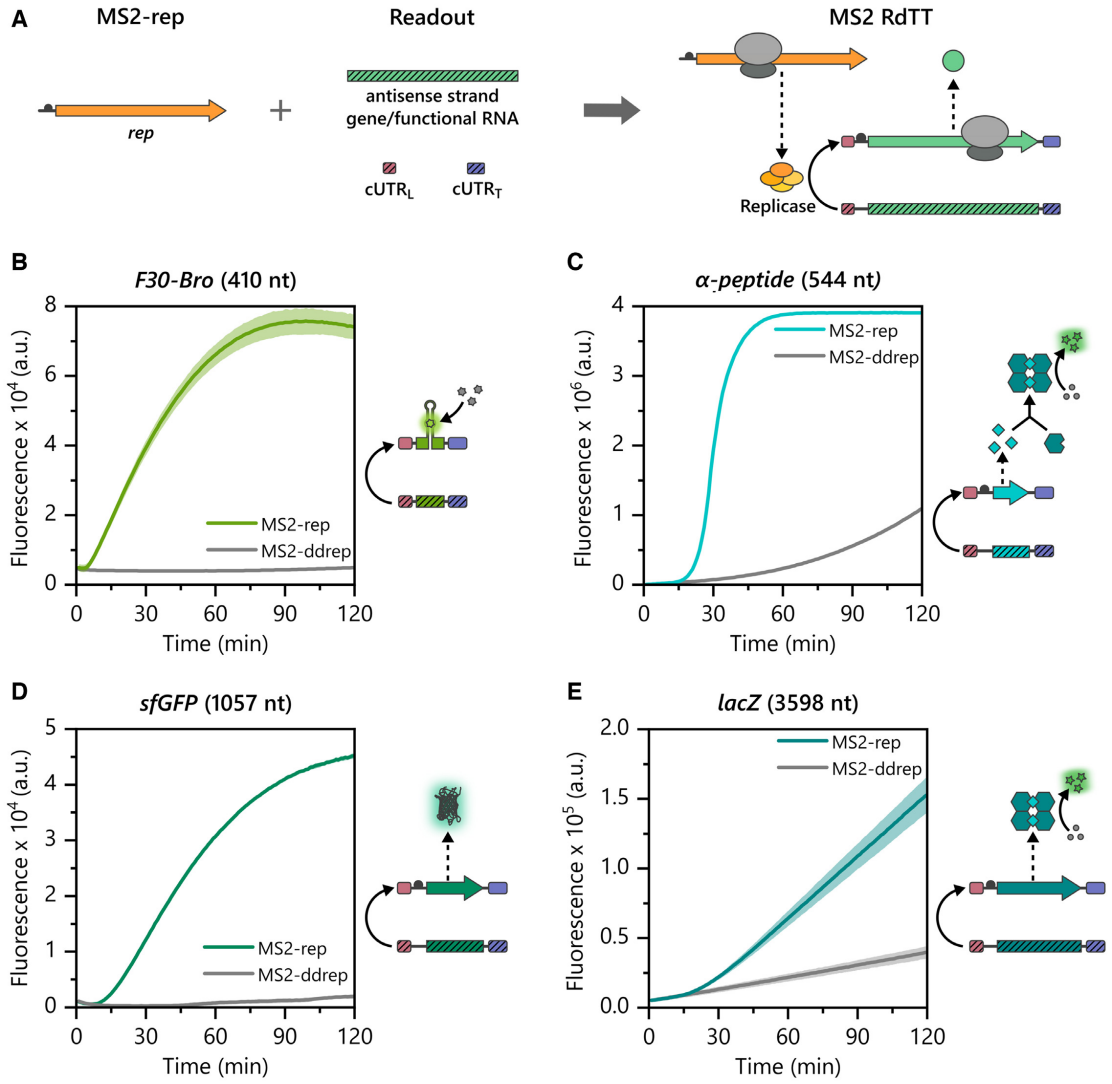
Terminal fusion of antisense strands with complementary UTRs (cUTRs) enables MS2 RNA-dependent transcription-translation (MS2 RdTT) during cell-free expression. (**A**) Antisense strands of coding RNAs can be embedded between the MS2 cUTRs to form [*gene*]_MS2_ (−) RNA readout modules. A MS2-rep (+) RNA replicase module encoding the rep β subunit allows *in situ* replicase expression. Both modules combined enable MS2 RdTT: upon expression of *rep* and formation of the replicase, [*gene*]_MS2_ (−) RNAs are transcribed by the replicase into [*gene*]_MS2_ (+) RNAs, which can be translated. (**B**) MS2 replicase-dependent transcription of [F30-Bro]_MS2_ (+) RNA from [F30-Bro]_MS2_ (−) RNA (410 nt) causes fluorogenic DFHBI-1T binding by the aptamer domain of [F30-Bro]_MS2_ (+). Time traces are from PURE reactions programmed with 350 nM [F30-Bro]_MS2_ (−) RNA and either 70 nM MS2-rep (+) RNA (light green) or MS2-ddrep (+) RNA (grey) encoding an inactive rep β subunit. (**C**) MS2 RdTT of the LacZ α-peptide enables α-complementation of the inactive ω-peptide resulting in the formation of active β-galactosidase (β-gal) tetramer, which catalyses fluorogenic hydrolysis of Fluorescein di-β-D-galactopyranoside (FDG). Fluorescence time traces are from PURE reactions programmed with 70 nM [*α*]_MS2_ (−) RNA (544 nt) and either 70 nM MS2-rep (+) RNA (cyan) or MS2-ddrep (+) RNA (grey). Note that the background hydrolysis in presence of MS2-ddrep (+) RNA is a result of residual impurities and/or residual β-gal activity by the ω-peptide. (**D**) MS2 RdTT of sfGFP using the 1057 nt [*sfGFP*]_MS2_ (−) RNA as input. Fluorescence time traces are from PURE reactions programmed with 70 nM [*sfGFP*]_MS2_ (−) RNA and either 70 nM MS2-rep (+) RNA (green) or MS2-ddrep (+) RNA (grey). (**E**) MS2 RdTT of full-length LacZ using the 3598 nt [*lacZ*]_MS2_ (−) RNA as input. Fluorescence time traces of β-gal catalysed FDG turnover in PURE reactions containing 70 nM [*lacZ*]_MS2_ (−) RNA and either 70 nM MS2-rep (+) RNA (dark cyan) or MS2-ddrep (+) RNA (grey). All experiments were performed in technical triplicates. The means ± SD were plotted but are not visible in some experiments due to the small deviations between the replicates.

To test this hypothesis, we created two types of RNA modules: Readout modules for the detection of RNA-dependent RNA transcription and a replicase module (MS2-rep (+) RNA) encoding the open reading frame of the rep β subunit. The readout modules were designed as such that they should serve as synthetic (−) RNA templates for the MS2 replicase and were created by inserting the antisense strands of genes between both cUTRs (Figure 3A). The expression of the rep β subunit directly from MS2-rep (+) RNA allowed us to bypass conventional T7 DdTT and made the system completely DNA-independent. This not only prevented competition of the T7 pol for NTPs, but also enabled a better control over the expression levels of the rep β subunit.

Both modules combined enabled MS2-based RNA-dependent transcription-translation (MS2 RdTT) in the PURE system (Figure 3A). In detail, we monitored (+) strand transcription of differently sized (m)RNAs from their respective (−) strand constructs using *de novo* synthesized MS2 replicase and, if applicable, their translation into functional proteins. As first readout, we co-incubated the PURE components with the replicase module MS2-rep (+) RNA, the fluorophore DFHBI-1T and the readout module [F30-Bro]_MS2_ (−) RNA, in which the antisense strand of the F30-Broccoli aptamer (F30-Bro) (46,47) was embedded between both cUTRs. We anticipated that a successful RNA-dependent RNA transcription of the 410 nt F30-Bro aptamer by the MS2 replicase would generate a fluorescent signal upon binding of the otherwise nonfluorescent DFHBI-1T. Indeed, we detected a strong increase in fluorescence just after several minutes of incubation at 37°C, confirming that the *in situ* expressed rep β subunit forms the MS2 replicase together with the host factors provided in the PURE system (Figure 3B). In contrast, F30-Bro aptamer transcription, i.e. fluorescence, was not detectable in presence of an RNA template encoding the catalytically inactive rep β subunit variant (D341S/D342V, MS2-ddrep (+) RNA), in which crucial residues for Mg^2+^ coordination in the conserved palm domain of the replicase had been mutated (17,48).

As second readout, we probed MS2 RdTT-dependent α-complementation of the *E. coli* β-galactosidase (49). Here, a small N-terminal fragment of the β-galactosidase (α-peptide) complements the otherwise catalytically inactive C-terminal ω-protein, thereby restoring β-galactosidase activity (β-gal, coding gene is *lacZ*). We programmed PURE reactions with [*α*]_MS2_ (−) RNA (544 nt), MS2-rep (+) RNA, recombinant ω-protein and the fluorogenic β-galactosidase substrate Fluorescein di-β-D-galactopyranoside (FDG). As expected, we observed strong FDG turnover suggestive of successful α-complementation of the active β-gal tetramer (Figure 3C). Similarly, we observed successful MS2 RdTT of super folder green fluorescent protein (50) (sfGFP, 1057 nt, Figure 3D) as well as full-length *lacZ* mRNA (3598 nt, Figure 3E), which is even longer than the entire wild-type MS2 genome. During the course of these experiments, we also found that the addition of molecular crowders such as PEG or Ficoll increases protein yields considerably (Supplementary Figure S1). For this reason, subsequent MS2 RdTT experiments were conducted in presence of 6% (w/v) PEG 4000 unless stated otherwise.

*In vitro* transcription-translation reactions can be subject to considerable variabilities reflecting in part the complexity of translation, variabilities in template folding and batch-to-batch variations of the transcription-translation machinery (51,52). To assess the reproducibility of the MS2 RdTT system, we performed batch-to-batch variation experiments. To this end, we programmed two different batches of the commercial PURExpress system with [*lacZ*-RBS_2_]_MS2_ (−) RNA, FDG and either MS2-rep (+) RNA or MS2-ddrep (+) RNA. Minor differences between the two samples were indeed observable (Supplementary Figure S2), but these are in line with the multi-component reaction setup and the reported variations between different batches of the PURE system used (51,52).

In conclusion, we demonstrated that the *de novo* synthesized rep β subunit and translation factors present in the PURE system form an active replicase whose RNA-dependent RNA transcription activity can be detected *in situ* by MS2 RdTT using various engineered (−) RNA templates.

### MS2 UTRs enable stoichiometric MS2 RdTT

**Figure 4.**
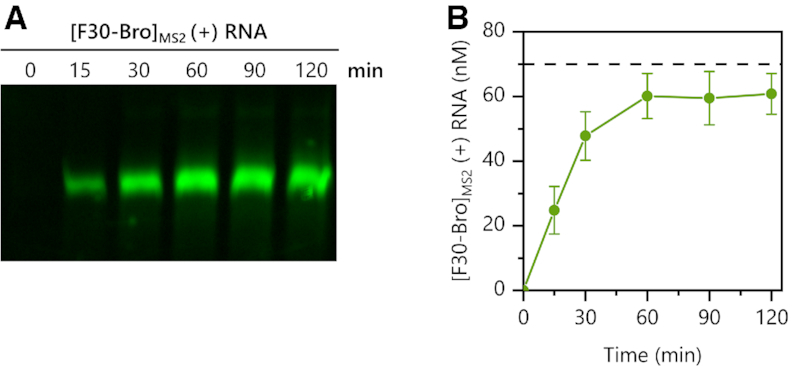
In-gel detection of transcribed [F30-Bro]_MS2_ (+) RNA in MS2 RdTT reactions. (**A**) A representative native PAGE gel showing transcription of the Broccoli aptamer by MS2 replicase after DFHBI-1T staining. The PURE reaction was programmed with 70 nM [F30-Bro]MS2 (−) RNA and 70 nM MS2-rep (+) and sampled over time. (**B**) Quantification of *de novo* synthesized [F30-Bro]_MS2_ (+) RNA shown in (A). The dashed line represents the initial [F30-Bro]_MS2_ (−) RNA input concentration and suggests stoichiometric (+) strand synthesis by the MS2 replicase under the given experimental conditions. [F30-Bro]_MS2_ (+) RNA concentrations were estimated using a standard curve derived from fluorescence band intensities of known input amounts of [F30-Bro]_MS2_ (+) RNA (Material and Methods). The experiment was performed in technical duplicates. The means ± SD were plotted.

After demonstrating successful (−) to (+) ssRNA conversion by the MS2 replicase, we sought to determine the amount of (+) strand that is synthesized during a typical RdTT reaction and whether the *de novo* synthesized (+) strand would also serve as template for more (−) strand synthesis, thereby initiating an RNA amplification cycle. To quantify the amount and kinetics of the *de novo* transcribed (+) strand, we monitored the fluorescence increase upon [F30-Bro]_MS2_ (+) RNA transcription in MS2 RdTT reactions programmed with [F30-Bro]_MS2_ (−) RNA and MS2-rep (+) at different time points of the reaction by non-denaturing PAGE (Figure 4A). The native state PAGE showed a single defined band suggesting that once template binding and initiation have successfully occurred, the processivity of MS2 replicase is very high. The total concentration of F30-Bro aptamer synthesized during MS2 RdTT was determined by comparing the integrated band intensities with standards of known input amounts of [F30-Bro]_MS2_ (+) RNA (Material and Methods). When PURE reactions were programmed with 70 nM [F30-Bro]_MS2_ (−) RNA and 70 nM MS2-rep (+), we observed a near-stoichiometric formation of full-length [F30-Bro]_MS2_ (+) RNA during the first hour of incubation (Figure 4B). No further synthesis or even amplification was observed during longer incubation times, which is suggestive of either inactivation of the MS2 replicase complex, consumption of NTP pools, or the sequestration of [F30-Bro]_MS2_ (−) template RNA into inert RNA duplexes (33).

To further probe whether MS2 replicase is capable of synthesizing (−) strands from engineered (+) strands, we initiated RdTT reactions directly with *in vitro* transcribed [F30-Bro]_MS2_ (+) RNA. If both (−) and (+) strand could serve as a template, a further increase in F30-Bro levels would have been expected. However, no significant fluorescence increase compared to a negative control was observed during 3 h of incubation, implying that (+) strand readout modules do not serve as templates for (−) strand synthesis under our tested conditions (Supplementary Figure S3).

In the following, we tested if the amount of [F30-Bro]_MS2_ (+) RNA could be increased by adding more (−) strand RNA template. To this end, we titrated different concentrations of [F30-Bro]_MS2_ (−) RNA into MS2 RdTT reactions containing 70 nM MS2-rep (+) and quantified the reactions after 2 h of incubation (Supplementary Figure S4A). Under these conditions, the amount of *de novo* synthesized [F30-Bro]_MS2_ (+) RNA increased until 210 nM input [F30-Bro]_MS2_ (−) RNA and plateaued at a maximal yield of ∼90 nM (Supplementary Figure S4B). Higher amounts of input template reduced the yields of overall (+) strand, suggesting that the excess amount of free (−) strand promotes the formation of non-fluorescent dead-end duplexes by enhanced annealing with newly synthesized [F30-Bro]_MS2_ (+) RNA.

In conclusion, the new MS2 RdTT system enables unidirectional conversion from (−) to (+) strand of various RNAs without further (unregulated) amplification as in most current Qβ systems (24,27). The lack of amplification in the engineered RNA results presumably from the absence of RNA motifs required for (+) RNA recognition and initiation by the replicase. Indeed, for the related Qβ phage, (−) RNA transcription from genomic (+) RNA is highly dependent on internal RNA domains, long-range RNA–RNA and specific RNA–protein interactions (9,53).

### Only the cUTR_L_ domain is required for MS2 RdTT of (−) strand RNA

In an attempt to further minimize the UTRs required for MS2 RdTT, we compared MS2 RdTT-based α-complementation with the α-peptide mRNA embedded between either full-length cUTRs (Supplementary Figure S5A) or minimized MS2 cUTRs consisting of only the terminal hairpins of the MS2 genome (Supplementary Figure S5B). When using these shortened constructs, formation of the active β-gal tetramer was strongly reduced (Supplementary Figure S5B), implying that the minimized RNA domains are only poorly recognized by the replicase.

**Figure 5.**
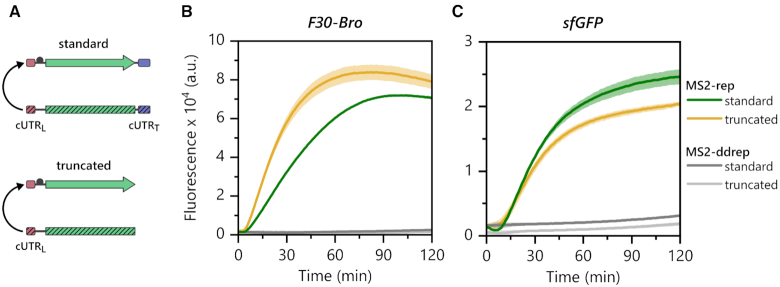
The MS2 replicase only requires the cUTR_L_ for MS2 RdTT. (**A**) The (−) strand RNA constructs for MS2 RdTT were either ‘standard’ reporter modules containing both cUTR_L_ and cUTR_T_ domains or truncated modules lacking the cUTR_T_ domain. (**B**) Fluorescence time traces of PURE reactions programmed with 70 nM MS2-rep (+) RNA and either 350 nM standard [F30-Bro]_MS2_ (−) RNA (green) or truncated [F30-Bro]_MS2_ (−) RNA (yellow). The two corresponding negative controls using MS2-ddrep (+) RNA are shown in grey. (**C**) Fluorescence time traces of PURE reactions setup with 70 nM MS2-rep (+) RNA and either 70 nM standard [*sfGFP*-RBS_2_]_MS2_ (−) RNA (green) or truncated [*sfGFP*-RBS_2_]_MS2_ (−) RNA (yellow). Negative controls using MS2-ddrep (+) RNA are shown in grey. The experiments were performed in technical triplicates. The means ± SD are displayed, except for that of the negative control reaction containing standard [F30-Bro]_MS2_ (−) RNA and MS2-ddrep (+) RNA, which was calculated from duplicates. SDs are not visible in some experiments due to the small deviations between the replicates.

Next, we set out to test if both cUTRs of (−) strand templates are required for MS2 RdTT. For the Qβ replicase, it has been suggested that both 5′ and 3′ termini of genuine RNA templates cooperate during and after the initiation step, presumably via a circular configuration through a terminal helix (44). However, when we compared the levels of F30-Broccoli aptamer transcribed from a standard [F30-Bro]_MS2_ (−) RNA (containing both full-length cUTRs) with transcription from a truncated [F30-Bro]_MS2_ (−) RNA lacking the cUTR_T_ domain (Figure 5A), we found that transcription from the truncated template occurred at a higher rate compared to the full-length construct (Figure 5B). On the contrary, deleting the cUTR_T_ from a longer sfGFP construct ([*sfGFP*-RBS_2_]_MS2_ (−) RNA) had barely any impact on sfGFP expression levels (Figure 5C). Both findings imply that the cUTR_T_ domain is not essential for MS2 RdTT. The observed differences between 5′-terminally truncated and full-length (−) strand templates are rather suggestive of a context-dependent influence of the 129 nt segment on RNA template structure and/or stability rather than of a direct role of the RNA domain in transcription. For example, deleting the cUTR_T_ from the 410 nt F30-Broccoli construct decreases the overall RNA length and, thus, the synthetic burden for transcription by ∼43%, which may explain the overall increase in synthesis yields by MS2 replicase.

### The MS2 RdTT system is active in cell-sized emulsion droplets

**Figure 6.**
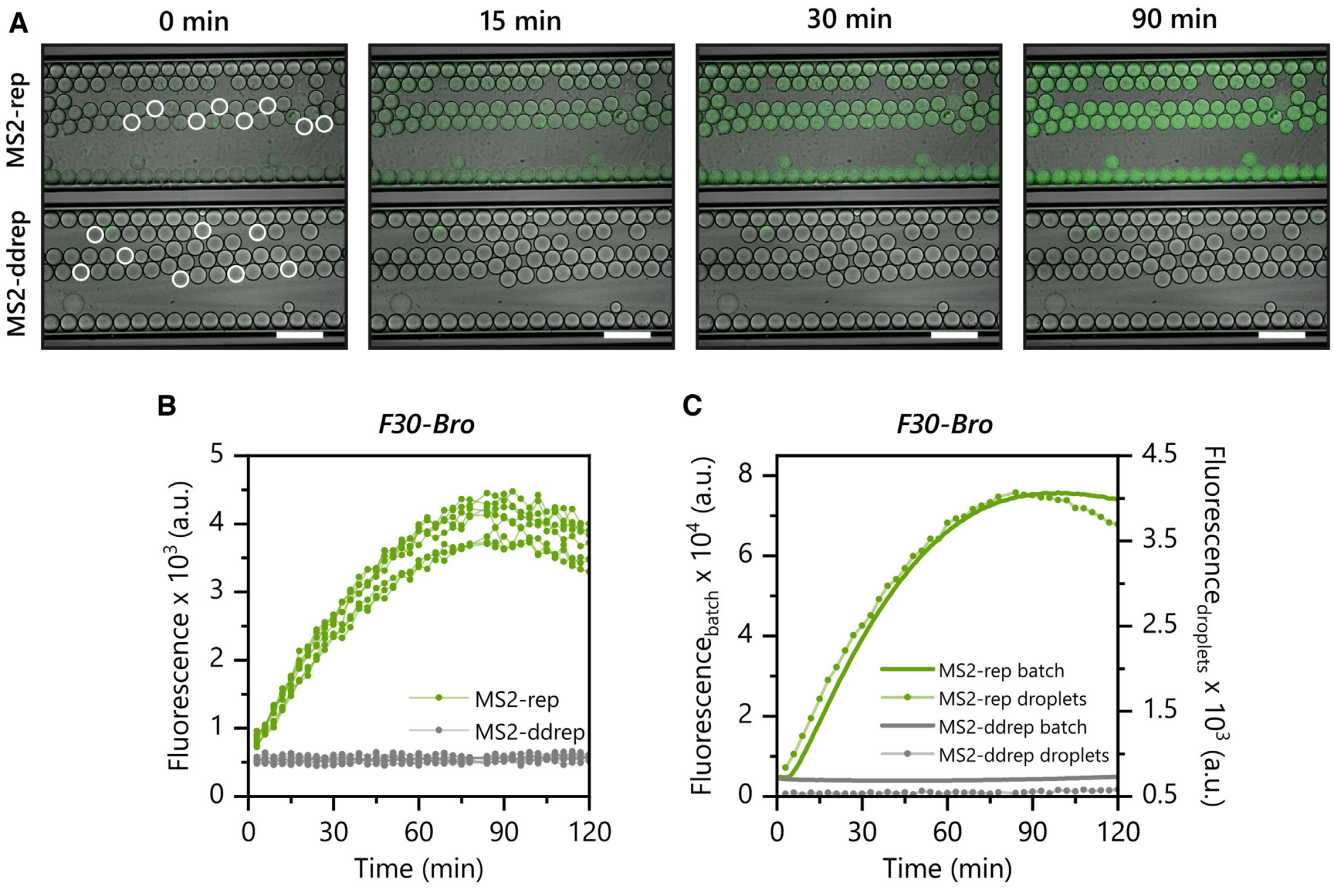
MS2 RdTT is compatible with microfluidic encapsulation in water-in-oil emulsion droplets. (**A**) Micrographs of a representative section of glass capillaries containing droplets enclosing PURE reactions expressing 350 nM [F30-Bro]_MS2_ (−) RNA and either 70 nM MS2-rep (+) RNA (upper capillary) or MS2-ddrep (+) RNA (lower capillary). Fluorescence images were taken at the specified times during incubation at 37°C (λ_ex_ = 488 nm, λ_em_ = 559 nm). Shown are overlays of bright-field and fluorescence images (green). Scale bars are 200 μm. (**B**) Fluorescence signals from eight individual droplets (marked in (A)) encapsulating PURE reactions containing [F30-Bro]_MS2_ (−) RNA and either MS2-rep (+) RNA (light green) or MS2-ddrep (+) RNA (grey). (**C**) Comparison of reaction kinetics from droplets expressing [F30-Bro]_MS2_ (−) RNA and either MS2-rep (+) (light green dotted line, average from (B)) or the equivalent batch reactions (light green solid line). The corresponding negative controls containing MS2-ddrep (+) RNA and [F30-Bro]_MS2_ (−) RNA are shown in grey (dotted – droplets, solid – batch).

Compartmentalization of *in vitro* translation systems is a well-established method in synthetic biology and useful in applications such as high-throughput screening, molecular evolution of enzymes, or bottom-up synthesis of artificial cells (54–58). The MS2 RdTT system could be useful in these types of experiments such as the evolution of DNA-modifying enzymes where the presence of an encoding DNA-template in addition to the selection substrate might be incompatible with the optimal selection strategy. To test if MS2 RdTT can enable DNA-independent transcription also in μm-sized water-in-oil emulsion droplets formed from a biocompatible surfactant in a fluorinated oil, we encapsulated ice-cold PURE reaction samples containing [F30-Bro]_MS2_ (−) RNA and MS2-rep (+) RNA (or MS2-ddrep (+) RNA as control) using a custom microfluidic setup (for details see Material and Methods) and monitored the fluorescence of individual droplets by fluorescence microscopy during incubation at 37°C. We observed robust fluorogenic transcription of the F30-Broccoli aptamer in the droplets during the first 90 min (Figure 6A, B and Supplementary Movie S1). The averaged time traces of eight individual droplets showed similar kinetics as in the batch reaction (Figure 6C). We could also show MS2 RdTT of active full-length β-gal under the same conditions (Supplementary Figure S6A, B and Supplementary Movie S2), further demonstrating that both formation and activity of the MS2 replicase are compatible with emulsion-based compartmentalization and that the overall system is readily compatible with typical microencapsulation-based evolution protocols.

### The MS2 RdTT can be used to trigger DdTT

**Figure 7.**
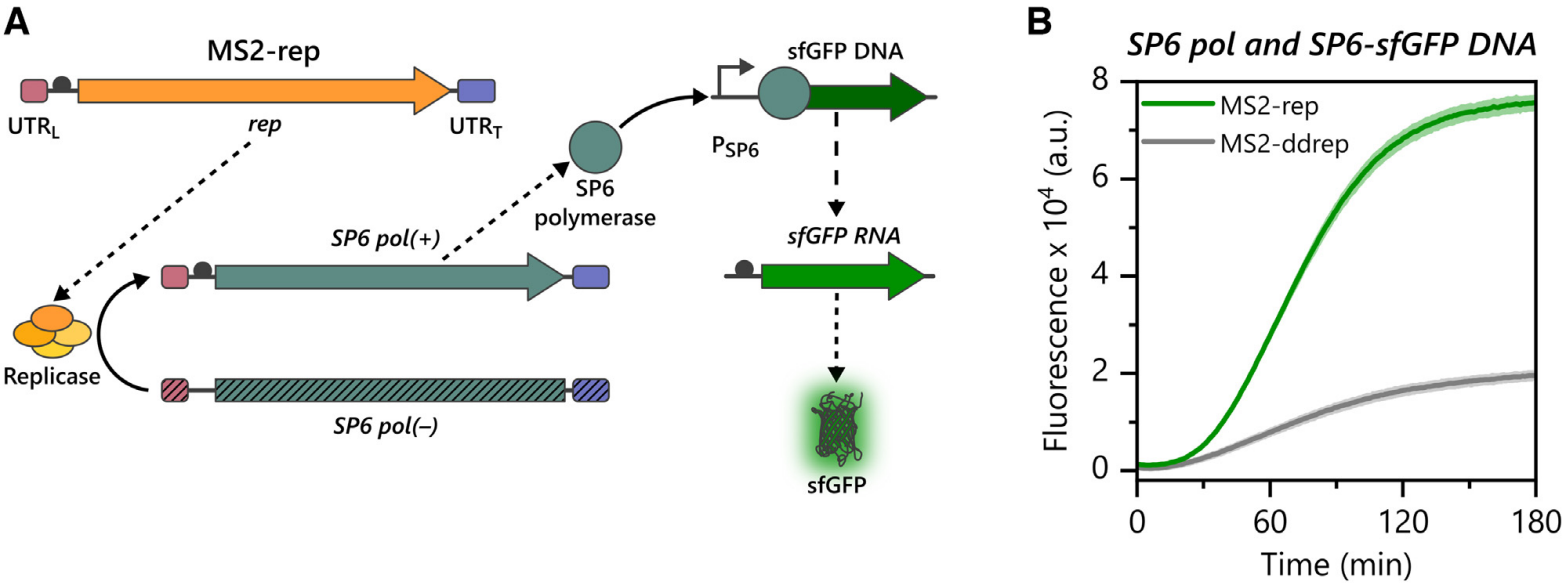
MS2 RdTT of SP6 pol enables coupling with SP6 DdTT. (**A**) Upon translation of MS2-rep, the formed replicase transcribes [*SP6 pol*]_MS2_ (−) RNA into the complementary sense strand. The latter is translated into SP6 pol, which accepts sfGFP DNA (SP6-sfGFP DNA) under the control of a SP6 promoter (P_SP6_) as template for DNA-dependent RNA transcription. Transcribed sfGFP mRNA is subsequently translated into the fluorescent sfGFP protein. (**B**) Fluorescence time traces of PURE reactions programmed with 70 nM MS2-rep (+) RNA or MS2-ddrep (+) RNA, 35 nM [*SP6 pol*]_MS2_ (−) RNA and 16 nM (125 ng) SP6-sfGFP DNA. MS2-ddrep (+) RNA was used as a negative control. The experiment was performed in technical triplicates. The means ± SD are displayed.

Having shown that the MS2 RdTT system enables complex schemes of coupled genetic/enzymatic information transfer, we wondered if we could use the system to control the otherwise orthogonal *in vitro* transcription-translation from DNA templates (i.e. DdTT). Such a ‘cross-talk’ between DNA- and RNA-dependent *in vitro* transcription-translation would largely expand the repertoire for the generation of synthetic circuits and switches used in synthetic biology. As a proof of concept, we designed an experiment such that the synthesis of active MS2 replicase would lead to MS2 RdTT of SP6 DNA-dependent RNA polymerase (SP6 pol) using [*SP6 pol*]_MS2_ (−) RNA as the template. The *de novo* expressed SP6 pol should then catalyse SP6 DdTT of sfGFP from a linear DNA template with an SP6 promoter (SP6-sfGFP DNA). We chose the SP6 pol for DdTT to bypass the T7 pol, which is already present in the PURE system, because both polymerases use different promotors. Altogether, the system coupling both MS2 RdTT and SP6 DdTT combines 5 steps of macromolecular synthesis in a one-pot reaction: Three translation events (rep β subunit, SP6 pol and sfGFP) and two transcription events ([*SP6 pol*]_MS2_ (+) RNA and sfGFP mRNA) (Figure 7A). To our delight, we were indeed able to switch on expression of sfGFP via MS2 RdTT of SP6 pol (Figure 7B). Thus, our new MS2 RdTT system can be directly used to trigger DdTT, thereby providing an additional control layer to the design of synthetic genetic circuits. In the current setup based on the commercially available PURE system, we observed some background sfGFP expression from leaky transcription of the SP6 promotor by the T7 pol present in the kit (Supplementary Figure S7). Such leakage could be omitted by using alternative, tailor-made PURE systems devoid of T7 pol (25,34,59–61).

## DISCUSSION

While MS2 replicase has been reported to be unstable and difficult to purify (9,10), we could show that the active replicase complex can be readily produced *in situ* in a recombinant *in vitro* transcription-translation system. The *in situ* produced replicase can directly initiate transcription of various RNA templates if its 3′ terminus is fused with cUTR_L_—a short 3′ terminal domain of the viral (−) strand. While low levels of RNA transcription can already be achieved with a minimalistic 43 nt version of cUTR_L_, maximal transcription levels require fusion to the full-length cUTR_L_ (124 nt, Supplementary Figure S5A, B). This implies that the complete cUTR_L_ domain contains additional structural elements for replicase template recognition and/or initiation.

In contrast to the transcription of (+) from (−) strands, we observed no (−) strand synthesis from synthetic (+) strand RNAs even if both UTRs were present. This finding implies either that the 3′-end of the tested (+) strand construct is only poorly accessible for replicase initiation or that additional sequence elements are required for a complete replication cycle. In agreement with the latter explanation, replication of the Qβ (+) strand by the Qβ replicase is crucially dependent on long-distance interactions between UTR_T_ (the 3′ terminal UTR of the (+) strand) and internal RNA-sites (9,62–64) and similar interactions were predicted to exist in the genomic MS2 (+) strand (64,65), which are missing in our engineered RNA templates. Alternatively, (+) to (−) strand replication might fail due to a potentially inaccessible 3′ end, which could prevent replication initiation. An additional yet unlikely possibility is that one or several yet unknown host factors are required for MS2 (−) RNA synthesis, which are missing in the PURE system. In general, the coordination of (−) strand synthesis and translation in ssRNA phages is a topologically complex process as both replicase and ribosomes compete for the same (+) RNA template but proceed with opposite polarities. Therefore, phages have established elaborate mechanisms to ensure that replication or translation are mutually exclusive to prevent collision events (9).

The ability of our system for stoichiometric (+) strand synthesis from an RNA template without further amplification distinguishes the MS2-based RdTT system from similar Qβ-based systems, which are currently the only other bacterial RNA-only *in vitro* transcription-translation systems described. In these systems, target genes are typically embedded in the (+) strands of small, non-genomic ‘parasitic’ RNA-scaffolds such as RQ135 (22,66) or MDV-1 (23,24,26). These extremely replication-competent RNAs lack the regulatory motifs required for the controlled timing and strength of gene expression and replication. Instead, they serve as templates for their own unregulated exponential amplification, which is limited only by either the formation of inert duplexes between (+) and (−) strand or the consumption of all available nucleotides. While RNA amplification is desirable in continuous evolution studies (25,27), it can be detrimental in experiments where resources are scarce, which for example is the case for *in vitro* transcription-translation batch reactions, or where the original coding template should remain unaltered. For example, during RNA amplification by the Qβ replicase, coding constructs are rapidly replaced by original non-coding parasite and strict compartmentalization and selective conditions are required to maintain the coding RNA pool (25,26). The MS2 RdTT system could therefore be useful for different applications in which RNA amplification is not required. The low fidelity of the MS2 replicase makes it suitable for direct *in vivo* or *in vitro* generation of RNA libraries for protein or aptamer selection from clonal RNA templates without the need for DNA mutagenesis. A study describing the *in vivo* use of Qβ replicase to generate mRNA libraries showed that the mutational spectrum of phage RNA replicases is close to the ideal (67). Furthermore, the independence of the MS2 RdTT system from DNA might increase the repertoire of selection strategies for DNA-modifying enzymes such as DNA nucleases, ligases, polymerases, recombinases or methyltransferases whose activity can interfere with a conventional DNA construct. As the MS2 RdTT system shows identical activity after encapsulation in water-in-oil emulsion droplets, the use of the system in *in vitro* selection protocols that are dependent on direct genotype-phenotype linkage through compartmentalization should be straightforward. Finally, we demonstrated that MS2 RdTT can be used to control conventional DdTT. This coupling of the two otherwise orthogonal expression systems could be used to expand the design repertoire of synthetic transcription-based genetic systems and circuits such as switches (68), oscillators (69,70), biosensors (71), or artificial multicellular systems (72).

## Supplementary Material

gkz817_Supplemental_Files
