# Supplementary material for: Cell-free expression of RNA encoded genes using MS2 replicase
Source: Nucleic Acids Res. 2019 Sep 30;47(20):10956–67. doi: 10.1093/nar/gkz817 (PMC6847885; doi:10.1093/nar/gkz817)
Supplement: gkz817_Supplemental_Files [file gkz817_supplemental_files.zip › Weise_et_al_SI.docx]

**Supplementary Data**

**Cell-free expression of RNA encoded genes using MS2 replicase**

Laura I. Weise^1^, Michael Heymann^2^, Viktoria Mayr^1^ and Hannes Mutschler^1,^*

^1^ Biomimetic Systems, Max Planck Institute of Biochemistry, Martinsried, 82152, Germany

^2^ Dept. Cellular and Molecular Biophysics, Max Planck Institute of Biochemistry, Martinsried, 82152, Germany

* To whom correspondence should be addressed. Tel: +49 89 8578 3420; Email: mutschler@biochem.mpg.de

**Contents**

Supplementary Figures S1-S7

Supplementary Movies S1-S2

Supplementary Material and Methods

Supplementary Tables S1-S3

Supplementary References

**
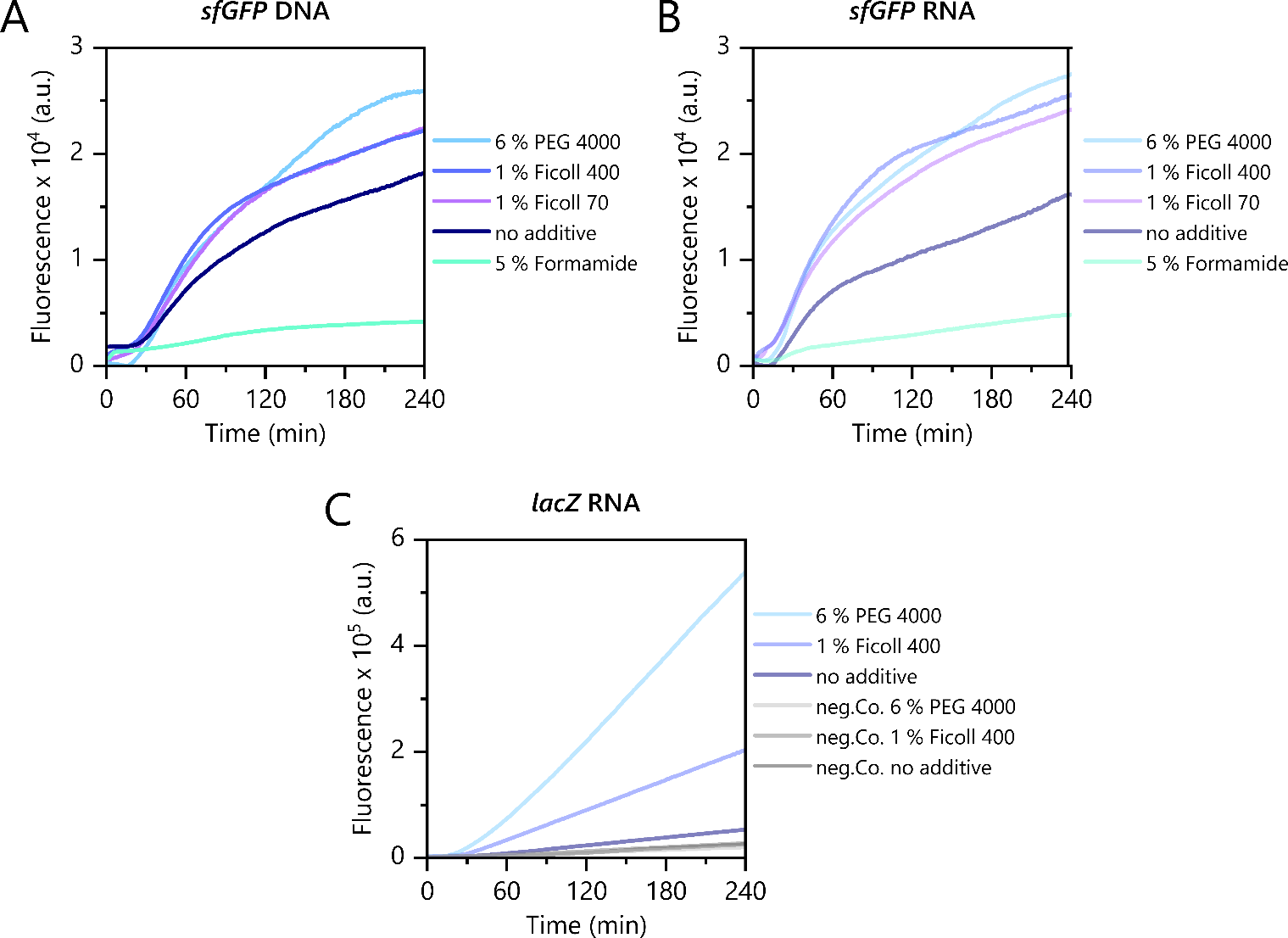
Supplementary Figure S1.** Effect of additives on MS2 RdTT reaction yields. **(A)** Fluorescence time traces of PURE reactions programmed with [*sfGFP*-RBS_2_]_MS2_ (-) RNA and Rep (+) DNA in presence of different additives (as indicated). **(B)** Equivalent time traces of PURE reactions programmed with [*sfGFP*-RBS_2_]_MS2_ (-) RNA and MS2-rep (+) RNA. **(C)** Fluorescence time traces of PURE reactions programmed with [*lacZ*-RBS_2_]_MS2_ (-) RNA, FDG and either MS2-rep (+) RNA or MS2-ddrep (+) RNA in presence of 6% (w/v) PEG 4000, 1% /w/v) Ficoll 400 or without additive. Please note that the ribosome binding site (RBS) for *sfGFP* and *lacZ* (RBS_2_) differs from the “standard” RBS (RBS_1_) of the *sfGFP* and *lacZ* constructs used in Figure 3D, E (Supplementary Methods and Supplementary Table S2).

**
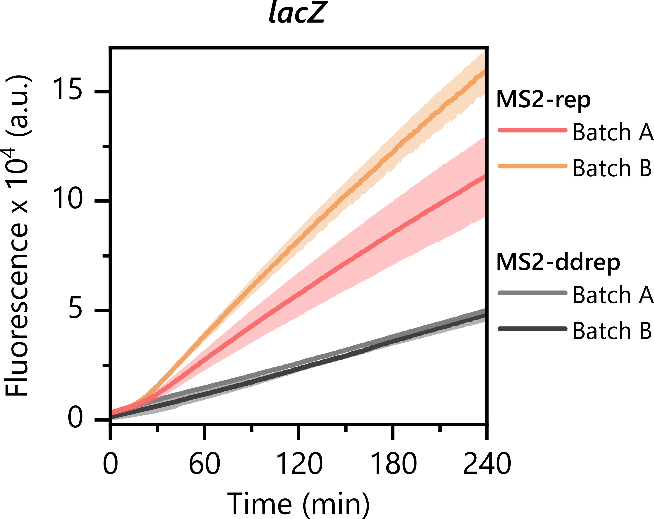
Supplementary Figure S2.** Batch-to-batch variations of MS2 RdTT. Two different batches of the commercial PURExpress system were used. Fluorescence time traces of PURE reactions programmed with [lacZ-RBS_2_]_MS2_ (-) RNA, FDG and either MS2-rep (+) RNA (orange or red) or MS2-ddrep (+) RNA (grey or black). All experiments were performed in technical triplicates. The means ± SD were plotted.


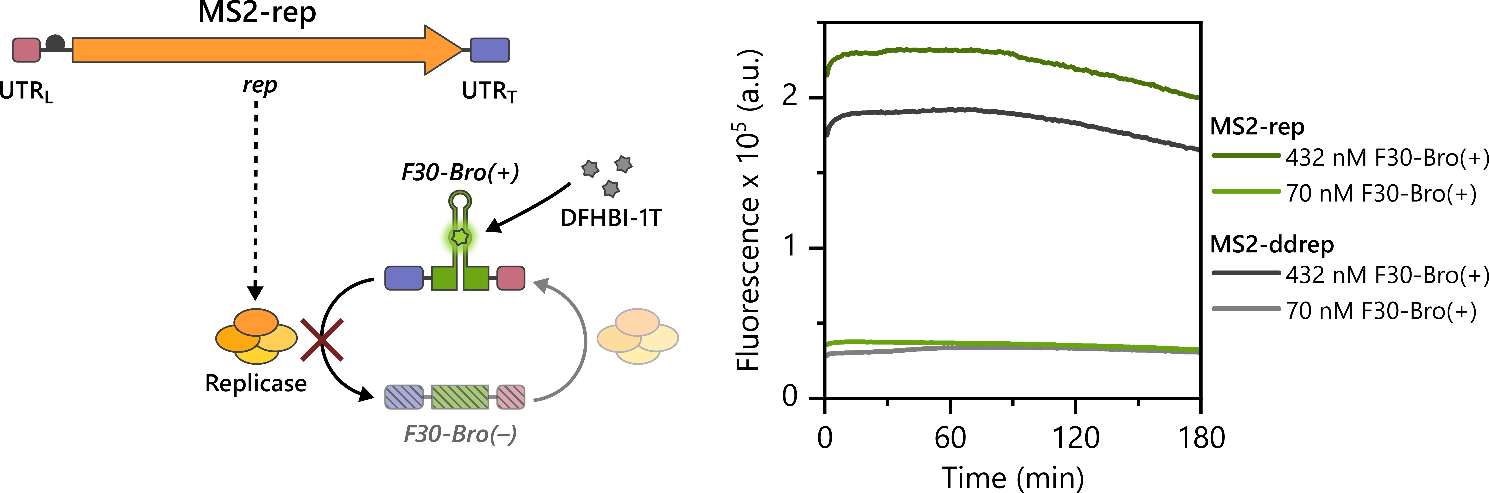
**Supplementary Figure S3.** The MS2 replicase is not able to replicate [F30-Bro]_MS2_ (+) RNA via a (-) RNA intermediate. The PURE reactions were programmed with MS2-rep (+) RNA or MS2-ddrep (+) RNA, DFHBI-1T and [F30-Bro]_MS2_ (+) RNA, which causes a fluorescent offset already at t = 0. Continuous synthesis of additional [F30-Bro]_MS2_ (+) RNA from *de novo* transcribed [F30-Bro]_MS2_ (-) RNA by the replicase would cause a time-dependent increase in DFHBI-1T fluorescence (left panel). However, PURE reactions producing active MS2 replicase (from MS2-rep (+) RNA, light and dark green) show no substantial increase in fluorescence relative to initial levels in reactions programmed with MS2-ddrep (+) RNA (grey and black) (right panel).

**
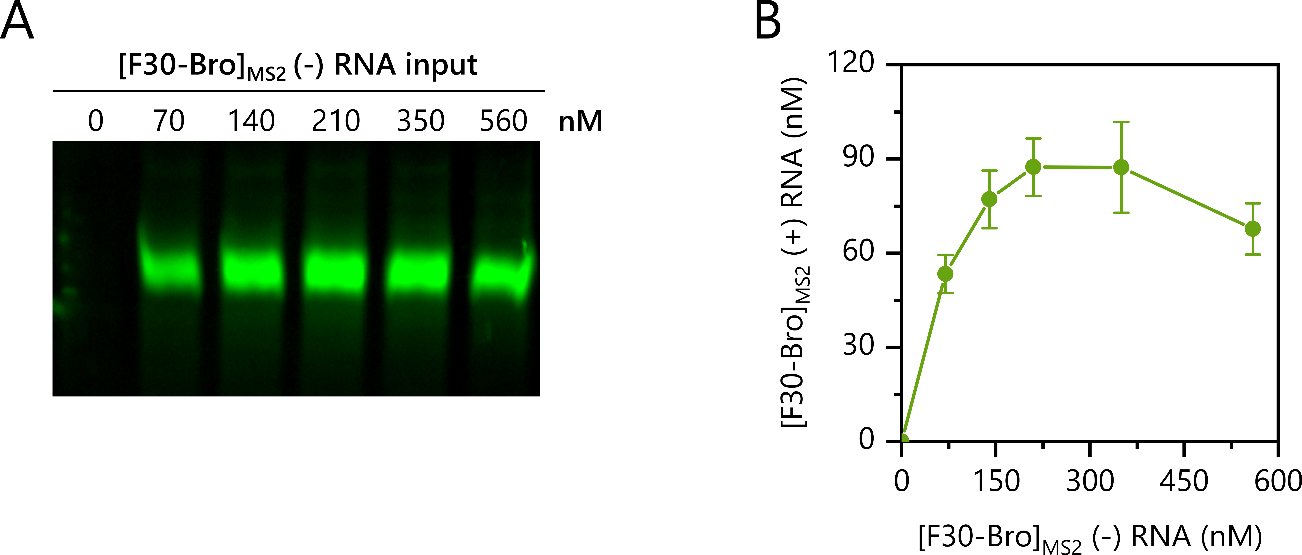
Supplementary Figure S4.** Effect of different input concentrations of [F30-Bro]_MS2_ (-) RNA on F30-Bro]_MS2_ (+) RNA yields under standard MS2 RdTT conditions. **(A)** *De novo* transcription of Broccoli aptamer by MS2 replicase was visualised by DFHBI-1T gel-staining after native TBE-PAGE. The PURE reaction was programmed with varying concentrations of [F30-Bro]_MS2_ (-) RNA (as indicated) and 70 nM MS2-rep (+) and incubated for 2 h at 37 °C. **(B)** Quantification of *de novo* synthesised [F30-Bro]_MS2_ (+) RNA shown in (A). [F30-Bro]_MS2_ (+) RNA concentrations were estimated using a standard curve derived from fluorescence band intensities of known input amounts of [F30-Bro]MS2 (+) RNA (Supplementary Files). The experiment was performed in technical triplicates. The means ± SD were plotted.

**
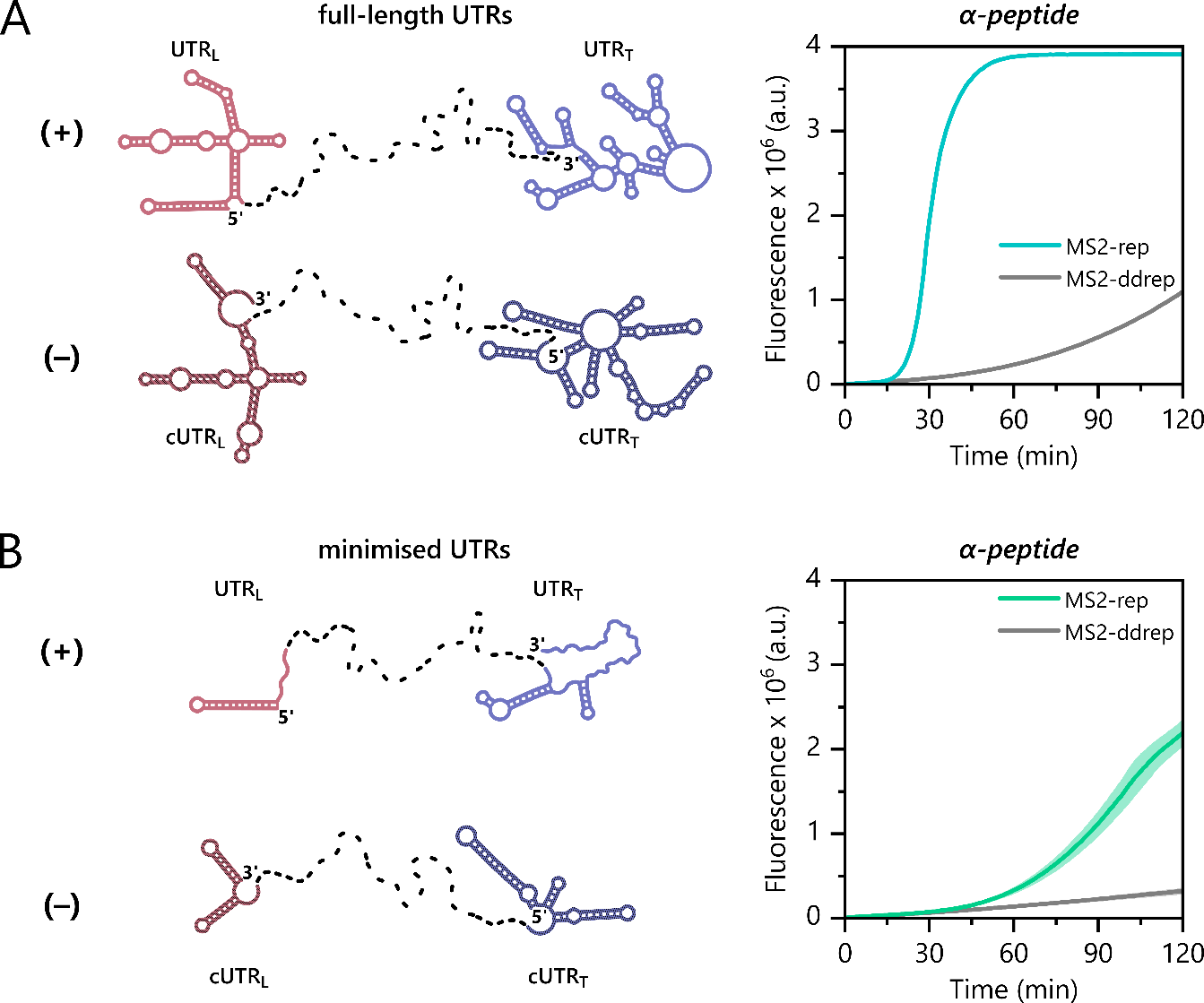
**

**Supplementary Figure S5.** Full-length MS2-derived cUTRs are required for efficient MS2 RdTT. (**A**) Left panel: Illustration of secondary structures of the full-length terminal domains in both (+) and (-) strand configuration. (+) strand structures are based on Dai *et al.* (1). (-) strand structures were predicted using RNAfold (2). Right panel: Fluorescence time traces of MS2 RdTT α-complementation reactions (same as in Figure 3C) programmed with [*α*]_MS2_ (-) RNA containing the full-length version of both cUTRs (cyan) and MS2-rep (+) RNA. A sample programmed equally but with MS2-ddrep (+) RNA is shown in grey. (**B**) Left panel: Illustration of secondary structures of the minimised terminal domains in both (+) and (-) strand configuration. Right panel: Fluorescence time traces of MS2 RdTT α-complementation reactions using [*α*]_minMS2_ (-) and either MS2-rep (+) RNA (spring green) or MS2-ddrep (+) RNA (grey) as the template. All experiments were performed in technical triplicates. The means ± SD are displayed.

**
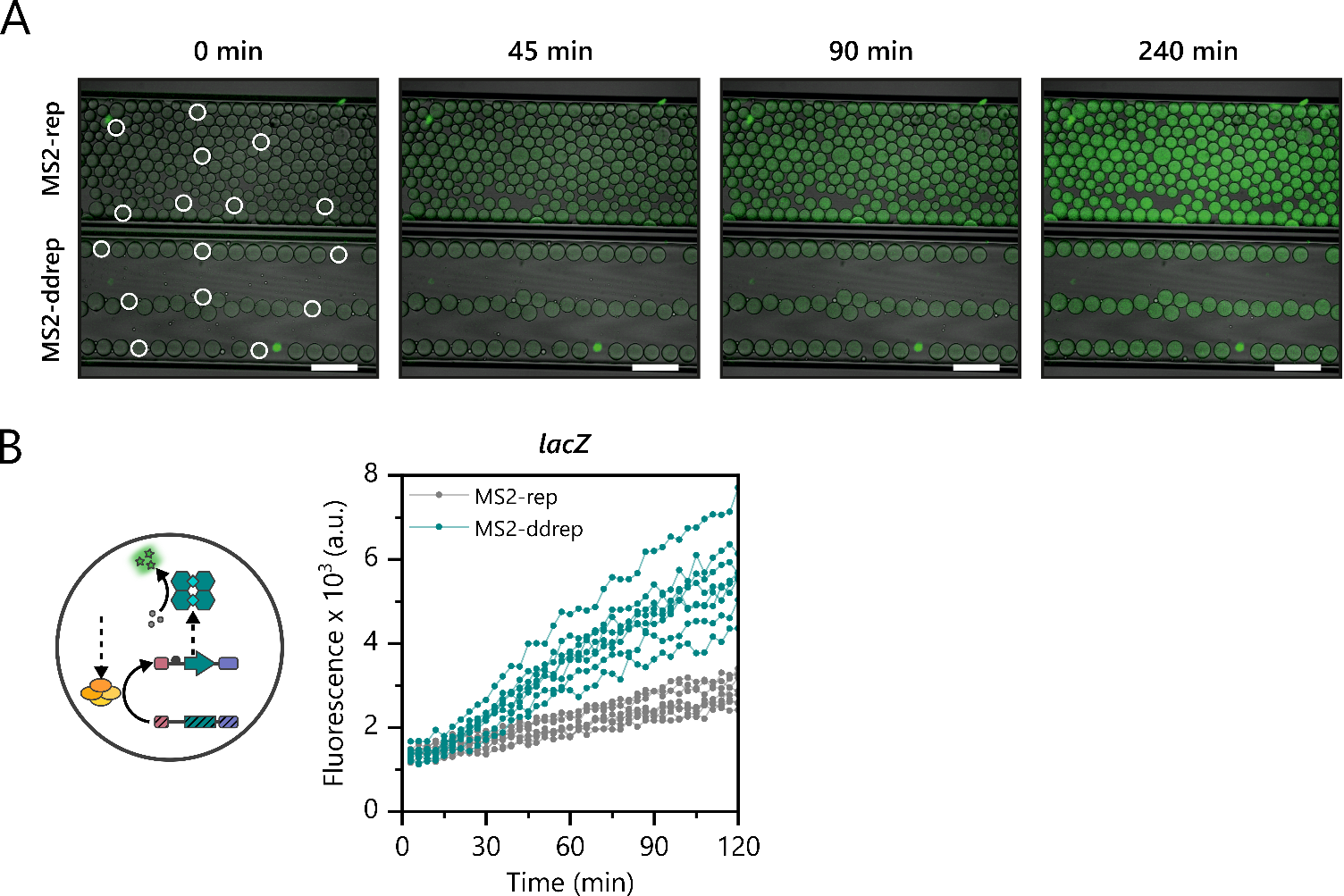
Supplementary Figure S6.** MS2 RdTT of full-length β-gal after microfluidic encapsulation. (**A**) Micrographs of a representative section of glass capillaries containing droplets enclosing PURE reactions programmed with [*lacZ*-RBS_2_]_MS2_ (-) RNA, FDG and either MS2-rep (+) RNA (upper capillary) or MS2-ddrep (+) RNA (lower capillary). Brightfield transmitted light images where overlaid with the fluorescence images colored green. Fluorescence images were recorded at the time points indicated during incubation at 37 °C (λ_ex_ = 488 nm, λ_em_ = 559 nm). The scale bars are 200 µm. (**B**) Schematic of the encapsulated fluorogenic reaction. The replicase catalyses transcription of [*lacZ*-RBS_2_]_MS2_ (+) RNA from [*lacZ*-RBS_2_]_MS2_ (-) RNA. The *de novo* expressed β-gal catalyses fluorogenic hydrolysis of the substrate FDG (left panel). Fluorescence signals from eight individual PURE-droplets (marked in (A)) programmed with [*lacZ*-RBS_2_]_MS2_ (-) RNA and either MS2-rep (+) RNA (dark cyan) or MS2-ddrep (+) RNA (grey) (right panel). Please note, that the circle size of the droplets in the figure does not correspond with the circle size used for the fluorescence detection.

***
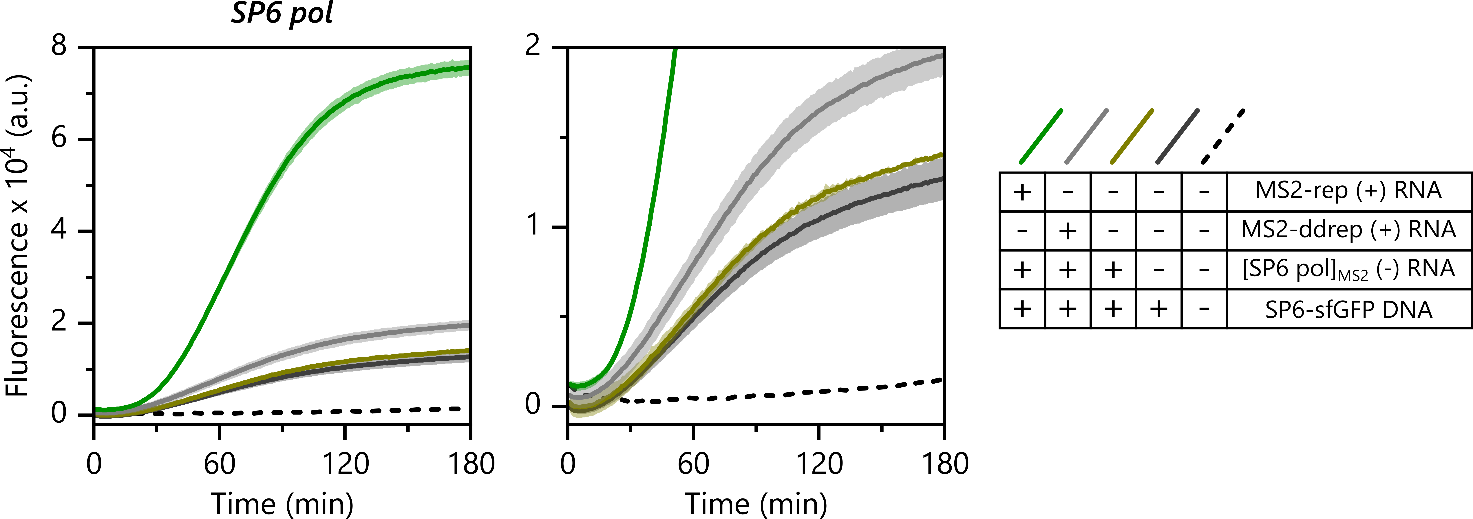
*Supplementary Figure S7.** Coupling of MS2 RdTT and SP6 DdTT and control reactions. Left panel: Fluorescence time traces shown in Figure 7 with additional controls where MS2 RdTT of SP6 pol had been omitted. Right panel: Zoom-in on the control reactions to emphasize leaky DdTT of SP6-sfGFP DNA by T7 pol included in the commercial PURExpress system. Control PURE reactions were programmed with [*SP6 pol*]_MS2_ (-) RNA and SP6-sfGFP DNA (olive), SP6-sfGFP DNA alone (dark grey) or [*SP6 pol*]_MS2_ (-) RNA, SP6-sfGFP DNA and MS2-ddrep (+) RNA (light grey). All three control reactions show weak levels of sfGFP background expression despite the absence of MS2 RdTT producing active SP6 pol. This implies leaky but weak T7 DdTT of sfGFP from the SP6 promotor. The two control reactions without MS2-ddrep (+) RNA (olive and dark grey) were performed in technical duplicates. The means of the triplicates or duplicates ± SD are displayed. A single control trace for the PURE reaction without any template was included (black line dashed).

**Supplementary Movies**

Supplementary Movie S1. Complete time-series of the F30-Broccoli fluorescence droplets shown in Figure 6. A single image after 27 cycles was excluded due to high external background fluorescence.

Supplementary Movie S2. Complete time-series of the β-gal fluorescence droplets shown in Supplementary Figure S6.

**Supplementary Material and Methods**

***Chemicals and oligonucleotides.*** The chemicals Ficoll®, Type 70 (Santa Cruz Biotechnology, sc-257529), FICOLL™ 400 (SERVA, 21373.01), Fluorescein di(β-D-galactopyranoside) (FDG, Santa Cruz Biotechnology, sc-221617), Formamide (Sigma Aldrich,47670-1L-F), Isopropyl β-D-1-thiogalactopyranoside (IPTG, Corning, 46-102-RF) and Polyethylene glycol 4000 (PEG 4000, SERVA, 33136.01) were used. 20 mM and 2 mM stock solutions of (Z)-4-(3,5-difluoro-4-hydroxybenzylidene)-2-methyl-1-(2,2,2-trifluoroethyl)-1H-imidazol-5(4H)-one (DFHBI-1T, Tocris, 5610) were prepared in Dimethylsulfoxid (DMSO, Carl Roth, A994.1). Further dilutions of DFHBI-1T from these stock solutions were prepared with nuclease-free water. DNA oligonucleotides were purchased from Integrated DNA Technologies (IDT) or Eurofins Genomics. All DNA oligonucleotide sequences are listed in Supplementary Table S1.

***Strains and media.*** All cloning was performed in Escherichia coli strain derivatives of DH5α (One Shot™ MAX Efficiency™ DH5α™-T1R, Thermo Fisher Scientific) and DH10β (NEB® 10-beta, NEB or Top10). Cells were grown in Lysogeny broth (LB-Lennox) in presence of carbenicillin (100 μg/mL, SERVA) or kanamycin (50 μg/mL, Corning) to maintain plasmids.

***Cloning of MS2-rep/MS2-ddrep.*** MS2 wild type RNA (Roche) was reverse transcribed with primer P1 (Supplementary Table S1) and the resulting cDNA was PCR-amplified with primer P2 (initially omitting the 5’ UTR i.e. UTR_L_) using the OneTaq® One-Step RT-PCR Kit (NEB). The dsDNA amplicon was sub-cloned into the pGEM®-T Easy backbone via TA cloning (pGEM®-T Easy Vector Systems, Promega) according to the manufacturer’s instructions. The resulting plasmid was further reduced to just encode the *rep* gene (encoding the replicase β subunit) and the UTR_T_. The initially missing UTR_L_ was derived from the α-peptide gBlock and inserted upstream of the *rep* gene via Gibson Assembly (GA) using the NEBuilder® HiFi DNA Assembly Master Mix (NEB) (Supplementary Table S3). The replication deficient (D341S/D342V) variant of the MS2 replicase β subunit (for ddRep DNA or MS2-ddrep) was created by site-directed mutagenesis of this plasmid using primers P3 and P4. The linear Rep DNA or ddRep DNA constructs were obtained by PCR amplification of the according plasmids using the primers P1 and P5.

***Cloning of MS2 RdTT (-) DNA templates.*** DNAs encoding [*α*]_MS2_ (+), [*sfGFP*]_MS2_ (+) and [*lacZ*]_MS2_ (+) (split into two fragments) were ordered as gBlocks® Gene Fragments from IDT. The full-length [*lacZ*]_MS2_ (+) was assembled from the two fragments with a 33 bp overlap. All inserts were cloned into pGEM-T Easy via isothermal GA using the Gibson Assembly® Master Mix (NEB) or the NEBuilder® HiFi DNA Assembly Master Mix (NEB). The coding sequence of the SP6 DNA-dependent RNA polymerase was PCR amplified from pBH176 (3) available in the lab and cloned into the MS2 scaffold by replacing sfGFP with SP6 pol ([*SP6 pol*]_MS2_ (+) with RBS_1_) using NEBuilder® HiFi DNA Assembly Master Mix (NEB). The α-peptide sequence embedded in the minimised MS2 scaffold ([*α*]_minMS2_) was ordered as synthetic gene in a pEX-A2 (Amp) vector from Eurofins Genomics. The DNA sequence for [F30-Bro]_MS2_ (+) encoding the F30-Broccoli RNA aptamer (4) was purchased as synthetic sequence in a pUCIDT (Amp) vector from IDT.

For sfGFP and lacZ, two constructs with two different RBS were generated. The original gBlocks contained a weaker RBS (RBS_2_, Supplementary Table S2), which was later changed into the stronger RBS_1_ by site-directed mutagenesis using primers (P6 and P7 or P6 and P8, respectively) with the Q5® Site-Directed Mutagenesis Kit (NEB). All constructs were verified by sequencing.

***Regular PCR*.** Standard PCRs for cloning or for the generation of MS2 RdTT (+) DNA were carried out with either the Q5® Hot Start High-Fidelity 2X Master Mix (NEB) or the Phusion® Hot Start Flex 2X Master Mix (NEB) according to the manufacturer’s instructions with template concentrations of 1 ng/µl reaction. To obtain MS2 RdTT (+) DNA templates for *in vitro* transcription the according plasmids and the primers P1 and P9 were used. The SP6-sfGFP DNA template was produced with the according plasmid and the primer P1 and P10.

***PCR-amplification of MS2 RdTT (-) DNA templates for in vitro transcription.*** PCR reactions for embedded antisense strands of the MS2 RdTT constructs were carried out with plasmid or linear DNA as template (1 ng/µl reaction) using the primer pairs P11 / P12 or P11 / P13 using the 2x Platinum™ SuperFi™PCR Master Mix (Thermo Fisher Scientific) according to the manufacturer’s instructions. Please note that 35 PCR cycles were necessary to obtain sufficient amounts of MS2 RdTT (-) DNA for *in vitro* transcription due to a generally low PCR efficiency during the amplification of the DNAs encoding (-) RNA strand templates. The primer pair P11 / P14 was used for PCR amplification of the shortened sfGFP construct ([*sfGFP*-RBS_2_]_MS2_ no cUTR_T_). The DNA template for the shortened F30-Bro construct ([F30-Bro]_MS2_ no cUTR_T_) was performed using 2x GoTaq® G2 Hot Start Green Master Mix (Promega) and the primer pair P11 / P15 according to the manufacturer’s instructions with template concentrations of 1 ng/µl reaction.

***In vitro transcription and RNA purification.*** RNAs were synthesized by run-off *in vitro* transcription using the TranscriptAid™ T7 High Yield Transcription Kit (Thermo Fisher Scientific) according to the manufacturer’s instructions. DNA templates were digested with TURBO™ DNase (Thermo Fisher Scientific) at 37 °C for 30 min. Reaction products were analysed by TAE/formamide RNA agarose gel electrophoresis (5) with SYBR Safe staining (Thermo Fisher Scientific). RNAs were purified using the RNeasy Mini Kit (Qiagen) according to the manufacturer’s instructions with slight modifications (before column binding 100 µL RLT buffer and 200 µL 2-propanol were added to 100 µl diluted transcription reaction). The concentration of RNAs was determined using absorbance measurements at 260 nm and OligoCalc (6). The concentration of [F30-Bro]_MS2_ (+) RNA and [F30-Bro]_MS2_ (-) RNA used in the gel quantification experiments (Figure 4 and Supplementary Figure S4) was determined using the Qubit ™ RNA BR Assay Kit (Thermo Fisher Scientific).

***Real-time fluorescence measurements of MS2 RdTT in Supplementary Figures****.* The standard PURE reaction mix was supplemented with templates encoding MS2 rep β subunit, MS2-RdTT (-) RNA templates and other necessary components and additives (final concentrations) as indicated in the following:

*sfGFP expression by MS2 RdTT (Supplementary Figure S1A):* 5 nM Rep DNA, 70 nM [*sfGFP*-RBS_2_]_MS2_ (-) RNA and 12 U RNase inhibitor (moloX) and as additives either 6% (w/v) PEG 4000, 1 % (w/v) Ficoll 400, 1% (w/v) Ficoll 70 or 5% (v/v) formamide. *sfGFP expression by MS2 RdTT (Supplementary Figure S1B):* 70 nM MS2-rep, 70 nM [sfGFP-RBS_2_]_MS2_ (-) RNA and 12 U RNase inhibitor (moloX) and as additive either 6% (w/v) PEG 4000, 1 % (w/v) Ficoll 400, 1% (w/v) Ficoll 70 or 5% (v/v) formamide. *β-gal expression by MS2 RdTT (Supplementary Figure S1C):* 70 nM MS2-rep / MS2-ddrep (+) RNA, 70 nM [*lacZ*-RBS_2_]_MS2_ (-) RNA, 4 U RNase inhibitor (moloX), 50 µM FDG substrate and as additive either 6% (w/v) PEG 4000 or 1 % (w/v) Ficoll 400. *Full-length β-gal expression by MS2 RdTT (Supplementary Figure S2):* 70 nM MS2-rep / MS2-ddrep (+) RNA, 70 nM [*lacZ*-RBS_2_]_MS2_ (-) RNA, 6% (w/v) PEG 4000 and 50 µM FDG substrate. *F30-Bro transcription by MS2 RdTT (Supplementary Figure S3):* 70 nM MS2-rep / MS2-ddrep (+) RNA, 432 nM or 70 nM [F30-Bro]_MS2_ (+) RNA, 6% (w/v) PEG 4000 and 10 µM DFHBI-1T dye. *β-gal α-complementation by MS2 RdTT (Supplementary Figure S5)*: 70 nM MS2-rep / MS2-ddrep (+) RNA, 70 nM [α]_MS2_ (-) RNA / 70 nM [α]_minMS2_ (-) RNA, 70 nM ω-protein and 50 µM FDG substrate. *SP6 pol expression by MS2 RdTT coupled with sfGFP expression by SP6 DdTT,* *13.5 µL total (Supplementary Figure S7):* 35 nM [*SP6 pol*]_MS2_ (-) RNA, 16 nM (125 ng) SP6-sfGFP DNA, 5,5% (w/v) PEG 4000 and 12 U RNase inhibitor (moloX) or 16 nM (125 ng) SP6-sfGFP DNA, 5,5% (w/v) PEG 4000 and 12 U RNase inhibitor (moloX).

***In-gel imaging and quantitation of in vitro transcribed [F30-Bro]_MS2_ (+) RNA.*** *Concentration-dependent analysis:* An appropriately up-scaled standard PURE reaction (~10-fold) was used as a master mix and programmed with 70 nM MS2-rep (+) RNA, 6% (w/v) PEG 4000 and 1U RNase inhibitor (NEB) per µl reaction (final reaction concentrations). Equal volumes of the master mix were supplemented with varying amounts of [F30-Bro]_MS2_ (-) RNA (0, 70, 140, 210, 350, 560 nM final concentration) and incubated for 2 h at 37 °C in nuclease-free PCR tubes (Thermo Fisher Scientific) using a ProFlex PCR System (Thermo Fisher Scientific). Following incubation, 5 µl sample aliquots were mixed with 5x native RNA loading buffer (50 mM Tris-HCl pH 8, 100 mM EDTA pH 8, 25% (v/v) glycerol, 0.05% (w/v) bromophenol blue) and shock-frozen in liquid nitrogen and stored at ‐80 °C until further use. The [F30-Bro]_MS2_ (+) RNA concentrations for the standard curve were 50, 100, 150, 250 and 450 nM (prepared as described in Material and Methods). Gel analysis and quantification was carried out as described in the Material and Methods section for the transcription kinetics of [F30-Bro]_MS2_ (+) RNA synthesis.

***Cloning, expression and purification of LacZΔM15.*** The sequence encoding LacZΔM15 (LacZ (b0344) Δ12-42) was cloned from the pSV-β-Galactosidase plasmid DNA (Promega) into a pET28a expression vector using blunt end cloning. The resulting fusion construct contained an N-terminal hexahistidine (His_6_)-tag, followed by a linker peptide (GGS_3_PR). Expression of LacZΔM15 was initiated by transformation of the expression plasmid into T7 Express Competent *E. coli* (High Efficiency, NEB) cells. LB (Lennox) medium supplemented with kanamycin was inoculated from a freshly grown overnight culture. Subsequently, cells were grown at 37 °C to an OD_600_ of ~0.6 and then induced by adding IPTG to a final concentration of 0.4 mM. For protein expression, cells were shifted to 15 °C for ~15 h and harvested by centrifugation (20 min, 10000 ×g, 4 °C). Cell pellets were resuspended in buffer A1 (50 mM Na_2_HPO_4_/NaH_2_PO_4_ pH 7.0, 300 mM NaCl, 10 mM imidazole) and lysed in a Homogenizer (EmulsiFlex-C3, Avestin). The lysate was clarified by centrifugation (1 h, 33,000 ×g, 4 °C) and filtering (0.45 µm) and loaded on a 1 ml His‐Trap HP (GE Healthcare) column pre-equilibrated in buffer A1. After a high salt wash with buffer A2 (50 mM Na_2_HPO_4_/NaH_2_PO_4_ pH 7.0, 1 M NaCl, 10 mM imidazole) the bound protein was eluted by applying a linear gradient with buffer A1 containing 500 mM imidazole. Fractions were pooled followed by a buffer-exchange step into buffer B1 (25 mM NaCl, 10 mM Tris-HCl pH 8.0) using an Econo-Pac 10DG column (Bio-Rad) according to the manufacturer’s instructions. Pooled protein fractions were bound to a Mono Q 5/50 GL column (GE Healthcare) equilibrated in buffer B1. After washing the resin with buffer B1 the bound protein was eluted with a linear gradient of 25 mM – 1 M NaCl. Fractions containing LacZΔM15 protein were concentrated to a final volume of 2 ml after buffer exchange to C1 (50 mM Na_2_HPO_4_/NaH_2_PO_4_ pH 7.0, 200 mM NaCl) using a centrifugal concentrator (Vivaspin 20 100 kDA MWCO, GE Healthcare). The concentrated protein was injected on a HiLoad Superdex 16/600 200 pg size‐exclusion column (GE Healthcare). LacZΔM15 eluted in two main peaks - one close to the exclusion volume (peak 1) and one peak at the expected size of a protein dimer (peak 2). The presence of highly purified amounts of LacZΔM15 in both peaks was verified by SDS-PAGE. α-complementation of β-galactosidase activity was successful for both peaks, but just the presumed dimeric species in peak 2 showed a lower background activity when performing *in vitro* transcription-translation complementation experiments with α-peptide RNA (+). Thus, only fractions from peak 2 were used in complementation experiments. Peak 2 was pooled, concentrated with a centrifugal concentrator (Vivaspin 20 100 kDA MWCO, GE Healthcare, ~40 μM) and aliquots shock-frozen in liquid nitrogen and stored at ‐80°C until further use. The protein concentration was determined spectroscopically using the calculated extinction coefficient at 280 nm. The protein was diluted before use with buffer R (5 mM Na_2_HPO_4_/NaH_2_PO_4_ pH 7.0, 20 mM NaCl).

**Supplementary Table S1.** Table of DNA oligonucleotides used in this study. T7 promoter sequences are underlined.

| **N^o^** | **5’-3’ sequence, mutations are in red** | **description** |
| --- | --- | --- |
| 1 | tgggtggtaactagccaagc | Primer UTR_T_ |
| 2 | acatgtcaggaacagttactgacg | Primer upstream of coat protein |
| 3 | ggcatctacgggagcgttattatatgccccagt | Mutagenesis primer MS2-rep (D341S/D342V), fw |
| 4 | actggggcatataataacgctcccgtagatgcc | Mutagenesis primer MS2-rep (D341S/D342V), rev |
| 5 | ttgtaatacgactcactataggccattcaaacatgagga | Primer upstream of rep protein with P_T7_ |
| 6 | tatacttaagcccaaacctcctaggaatggaattc | Mutagenesis primer for RBS_1_ sfGFP/lacZ, into UTR_L_ |
| 7 | aggaggaaaaaatatgagcaaaggagaagaact | Mutagenesis primer for RBS_1_ sfGFP, into gene |
| 8 | aggaggaaaaaatatgagcgaaaaatacatcgtc | Mutagenesis primer for RBS_1_ lacZ, into gene |
| 9 | ttgtaatacgactcactatagggtgggacccctttcgg | Primer UTR_L_ with P_T7_ |
| 10 | atttaggtgacactatagaagggcttaagtataaggaggaaaaaat | Primer upstream of sfGFP with P_SP6_ |
| 11 | gggtgggacccctttc | Primer cUTR_L_ |
| 12 | taatacgactcactatagggtggtaac | Primer cUTR_T_ with P_T7_, all other constructs |
| 13 | taatacgactcactatagggtggtaactagccaag | Primer cUTR_T_ with P_T7_, F30-Bro |
| 14 | taatacgactcactatagggagaaaacttaactatttgtagagctc | Primer sfGFP no cUTR_T_ with P_T7_ |
| 15 | taatacgactcactatagggttgccatgaatgatcccga | Primer F30-Bro no cUTR_T_ with P_T7_ |

**Supplementary Table S2*.*** Table of RBS used for MS2 RdTT (-) RNA constructs in this study (shown are the RBS-containing sequences between the UTR_L_ and the start codon, predicted RBS are shown in bold).

| **Name** | **5’-3’ sequence** | **description** |
| --- | --- | --- |
| RBS_1_ | gggcttaagtat**aaggag**gaaaaaat | Strong RBS, from PURExpress manual |
| RBS_2_ | cgggaaaacaattgtgagcggataacaatttcaca**caggaa**acagct | original RBS from *lacZ* |

**Supplementary Table S3.** Table of linear DNA constructs (used for in vitro RNA transcription if applicable). The displayed orientation is 5’-3’, the coding sequences are uppercase, mutations are shown in red, RBS were predicted. The overlap between the rep β subunit sequence and the UTR_T_ is shown in magenta.

| **Rep DNA (+) (T7 promoter-RBS-*rep*-UTR_T_)** |
| --- |
| **ttgtaatacgactcactataggccattcaaacatgaggattacccATGTCGAAGACAACAAAGAAGTTCAACTCTTTATGTATTGATCTTCCTCGCGATCTTTCTCTCGAAATTTACCAATCAATTGCTTCTGTCGCTACTGGAAGCGGTGATCCGCACAGTGACGACTTTACAGCAATTGCTTACTTAAGGGACGAATTGCTCACAAAGCATCCGACCTTAGGTTCTGGTAATGACGAGGCGACCCGTCGTACCTTAGCTATCGCTAAGCTACGGGAGGCGAATGATCGGTGCGGTCAGATAAATAGAGAAGGTTTCTTACATGACAAATCCTTGTCATGGGATCCGGATGTTTTACAAACCAGCATCCGTAGCCTTATTGGCAACCTCCTCTCTGGCTACCGATCGTCGTTGTTTGGGCAATGCACGTTCTCCAACGGTGCCTCTATGGGGCACAAGTTGCAGGATGCAGCGCCCTACAAGAAGTTCGCTGAACAAGCAACCGTTACCCCCCGCGCTCTGAGAGCGGCTCTATTGGTCCGAGACCAATGTGCGCCGTGGATCAGACACGCGGTCCGCTATAACGAGTCATATGAGTTTAGGCTCGTTGTAGGGAACGGAGTGTTTACAGTTCCGAAGAATAATAAAATAGATCGGGCTGCCTGTAAGGAGCCTGATATGAATATGTACCTCCAGAAAGGGGTCGGTGCCTTTATCAGACGCCGGCTCAAATCCGTTGGTATAGACCTGAATGATCAATCGATCAACCAGCGTCTGGCTCAGCAGGGCAGCGTAGATGGTTCGCTTGCGACGATAGACTTATCGTCTGCATCCGATTCCATCTCCGATCGCCTGGTGTGGAGTTTTCTCCCACCTGAGCTATATTCATATCTCGATCGTATCCGCTCACACTACGGAATCGTAGATGGCGAGACGATACGATGGGAACTATTTTCCACAATGGGAAATGGGTTCACATTTGAGCTAGAGTCCATGATATTCTGGGCAATAGTCAAAGCGACCCAAATCCATTTTGGTAACGCCGGAACCATAGGCATCTACGGGGACGATATTATATGCCCCAGTGAGATTGCACCCCGTGTGCTAGAGGCACTTGCCTACTACGGTTTTAAACCGAATCTTCGCAAAACGTTCGTGTCCGGGCTCTTTCGCGAGAGCTGCGGCGCGCACTTTTACCGTGGTGTCGATGTCAAACCGTTTTACATCAAGAAACCTGTTGACAATCTCTTCGCCCTGATGCTGATATTAAATCGGCTACGGGGTTGGGGAGTTGTCGGAGGTATGTCAGATCCACGCCTCTACAAGGTGTGGGTACGGCTCTCCTCCCAGGTGCCTTCGATGTTCTTCGGTGGGACGGACCTCGCTGCCGACTACTACGTAGTCAGCCCGCCTACGGCAGTCTCGGTATACACCAAGACTCCGTACGGGCGGCTGCTCGCGGATACCCGTACCTCGGGTTTCCGTCTTGCTCGTATCGCTCGAGAACGCAAGTTCTTCAGCGAAAAGCACGACAGTGGTCGCTACATAGCGTGGTTCCATACTGGAGGTGAAATCACCGACAGCATGAAGTCCGCCGGCGTGCGCGTTATACGCACTTCGGAGTGGCTAACGCCGGTTCCCACATTCCCTCAGGAGTGTGGGCCAGCGAGCTCTCCTCGGTAGctgaccgagggacccccgtaaacggggtgggtgtgctcgaaagagcacgggtccgcgaaagcggtggctccaccgaaaggtgggcgggcttcggcccagggacctccccctaaagagaggacccgggattctcccgatttggtaactagctgcttggctagttaccaccca** |
| **ddRep DNA (+) (T7 promoter-RBS-*rep*-UTR_T_)** |
| **ttgtaatacgactcactataggccattcaaacatgaggattacccATGTCGAAGACAACAAAGAAGTTCAACTCTTTATGTATTGATCTTCCTCGCGATCTTTCTCTCGAAATTTACCAATCAATTGCTTCTGTCGCTACTGGAAGCGGTGATCCGCACAGTGACGACTTTACAGCAATTGCTTACTTAAGGGACGAATTGCTCACAAAGCATCCGACCTTAGGTTCTGGTAATGACGAGGCGACCCGTCGTACCTTAGCTATCGCTAAGCTACGGGAGGCGAATGATCGGTGCGGTCAGATAAATAGAGAAGGTTTCTTACATGACAAATCCTTGTCATGGGATCCGGATGTTTTACAAACCAGCATCCGTAGCCTTATTGGCAACCTCCTCTCTGGCTACCGATCGTCGTTGTTTGGGCAATGCACGTTCTCCAACGGTGCCTCTATGGGGCACAAGTTGCAGGATGCAGCGCCCTACAAGAAGTTCGCTGAACAAGCAACCGTTACCCCCCGCGCTCTGAGAGCGGCTCTATTGGTCCGAGACCAATGTGCGCCGTGGATCAGACACGCGGTCCGCTATAACGAGTCATATGAGTTTAGGCTCGTTGTAGGGAACGGAGTGTTTACAGTTCCGAAGAATAATAAAATAGATCGGGCTGCCTGTAAGGAGCCTGATATGAATATGTACCTCCAGAAAGGGGTCGGTGCCTTTATCAGACGCCGGCTCAAATCCGTTGGTATAGACCTGAATGATCAATCGATCAACCAGCGTCTGGCTCAGCAGGGCAGCGTAGATGGTTCGCTTGCGACGATAGACTTATCGTCTGCATCCGATTCCATCTCCGATCGCCTGGTGTGGAGTTTTCTCCCACCTGAGCTATATTCATATCTCGATCGTATCCGCTCACACTACGGAATCGTAGATGGCGAGACGATACGATGGGAACTATTTTCCACAATGGGAAATGGGTTCACATTTGAGCTAGAGTCCATGATATTCTGGGCAATAGTCAAAGCGACCCAAATCCATTTTGGTAACGCCGGAACCATAGGCATCTACGGGAGCGTTATTATATGCCCCAGTGAGATTGCACCCCGTGTGCTAGAGGCACTTGCCTACTACGGTTTTAAACCGAATCTTCGCAAAACGTTCGTGTCCGGGCTCTTTCGCGAGAGCTGCGGCGCGCACTTTTACCGTGGTGTCGATGTCAAACCGTTTTACATCAAGAAACCTGTTGACAATCTCTTCGCCCTGATGCTGATATTAAATCGGCTACGGGGTTGGGGAGTTGTCGGAGGTATGTCAGATCCACGCCTCTACAAGGTGTGGGTACGGCTCTCCTCCCAGGTGCCTTCGATGTTCTTCGGTGGGACGGACCTCGCTGCCGACTACTACGTAGTCAGCCCGCCTACGGCAGTCTCGGTATACACCAAGACTCCGTACGGGCGGCTGCTCGCGGATACCCGTACCTCGGGTTTCCGTCTTGCTCGTATCGCTCGAGAACGCAAGTTCTTCAGCGAAAAGCACGACAGTGGTCGCTACATAGCGTGGTTCCATACTGGAGGTGAAATCACCGACAGCATGAAGTCCGCCGGCGTGCGCGTTATACGCACTTCGGAGTGGCTAACGCCGGTTCCCACATTCCCTCAGGAGTGTGGGCCAGCGAGCTCTCCTCGGTAGctgaccgagggacccccgtaaacggggtgggtgtgctcgaaagagcacgggtccgcgaaagcggtggctccaccgaaaggtgggcgggcttcggcccagggacctccccctaaagagaggacccgggattctcccgatttggtaactagctgcttggctagttaccaccca** |

| **MS2-rep (+) (T7 promoter-UTR_L_ -RBS-*rep*-UTR_T_)** |
| --- |
| **ttgtaatacgactcactatagggtgggacccctttcggggtcctgctcaacttcctgtcgagctaatgccatttttaatgtctttagcgagacgctaccatggctatcgctgtaggtagccggaattccattcctaggaggttttagacgccggccattcaaacatgaggattacccATGTCGAAGACAACAAAGAAGTTCAACTCTTTATGTATTGATCTTCCTCGCGATCTTTCTCTCGAAATTTACCAATCAATTGCTTCTGTCGCTACTGGAAGCGGTGATCCGCACAGTGACGACTTTACAGCAATTGCTTACTTAAGGGACGAATTGCTCACAAAGCATCCGACCTTAGGTTCTGGTAATGACGAGGCGACCCGTCGTACCTTAGCTATCGCTAAGCTACGGGAGGCGAATGATCGGTGCGGTCAGATAAATAGAGAAGGTTTCTTACATGACAAATCCTTGTCATGGGATCCGGATGTTTTACAAACCAGCATCCGTAGCCTTATTGGCAACCTCCTCTCTGGCTACCGATCGTCGTTGTTTGGGCAATGCACGTTCTCCAACGGTGCCTCTATGGGGCACAAGTTGCAGGATGCAGCGCCCTACAAGAAGTTCGCTGAACAAGCAACCGTTACCCCCCGCGCTCTGAGAGCGGCTCTATTGGTCCGAGACCAATGTGCGCCGTGGATCAGACACGCGGTCCGCTATAACGAGTCATATGAGTTTAGGCTCGTTGTAGGGAACGGAGTGTTTACAGTTCCGAAGAATAATAAAATAGATCGGGCTGCCTGTAAGGAGCCTGATATGAATATGTACCTCCAGAAAGGGGTCGGTGCCTTTATCAGACGCCGGCTCAAATCCGTTGGTATAGACCTGAATGATCAATCGATCAACCAGCGTCTGGCTCAGCAGGGCAGCGTAGATGGTTCGCTTGCGACGATAGACTTATCGTCTGCATCCGATTCCATCTCCGATCGCCTGGTGTGGAGTTTTCTCCCACCTGAGCTATATTCATATCTCGATCGTATCCGCTCACACTACGGAATCGTAGATGGCGAGACGATACGATGGGAACTATTTTCCACAATGGGAAATGGGTTCACATTTGAGCTAGAGTCCATGATATTCTGGGCAATAGTCAAAGCGACCCAAATCCATTTTGGTAACGCCGGAACCATAGGCATCTACGGGGACGATATTATATGCCCCAGTGAGATTGCACCCCGTGTGCTAGAGGCACTTGCCTACTACGGTTTTAAACCGAATCTTCGCAAAACGTTCGTGTCCGGGCTCTTTCGCGAGAGCTGCGGCGCGCACTTTTACCGTGGTGTCGATGTCAAACCGTTTTACATCAAGAAACCTGTTGACAATCTCTTCGCCCTGATGCTGATATTAAATCGGCTACGGGGTTGGGGAGTTGTCGGAGGTATGTCAGATCCACGCCTCTACAAGGTGTGGGTACGGCTCTCCTCCCAGGTGCCTTCGATGTTCTTCGGTGGGACGGACCTCGCTGCCGACTACTACGTAGTCAGCCCGCCTACGGCAGTCTCGGTATACACCAAGACTCCGTACGGGCGGCTGCTCGCGGATACCCGTACCTCGGGTTTCCGTCTTGCTCGTATCGCTCGAGAACGCAAGTTCTTCAGCGAAAAGCACGACAGTGGTCGCTACATAGCGTGGTTCCATACTGGAGGTGAAATCACCGACAGCATGAAGTCCGCCGGCGTGCGCGTTATACGCACTTCGGAGTGGCTAACGCCGGTTCCCACATTCCCTCAGGAGTGTGGGCCAGCGAGCTCTCCTCGGTAGctgaccgagggacccccgtaaacggggtgggtgtgctcgaaagagcacgggtccgcgaaagcggtggctccaccgaaaggtgggcgggcttcggcccagggacctccccctaaagagaggacccgggattctcccgatttggtaactagctgcttggctagttaccaccc** |
| **MS2-ddrep (+) (T7 promoter-UTR_L_ -RBS-*rep*-UTR_T_)** |
| **ttgtaatacgactcactatagggtgggacccctttcggggtcctgctcaacttcctgtcgagctaatgccatttttaatgtctttagcgagacgctaccatggctatcgctgtaggtagccggaattccattcctaggaggttttagacgccggccattcaaacatgaggattacccATGTCGAAGACAACAAAGAAGTTCAACTCTTTATGTATTGATCTTCCTCGCGATCTTTCTCTCGAAATTTACCAATCAATTGCTTCTGTCGCTACTGGAAGCGGTGATCCGCACAGTGACGACTTTACAGCAATTGCTTACTTAAGGGACGAATTGCTCACAAAGCATCCGACCTTAGGTTCTGGTAATGACGAGGCGACCCGTCGTACCTTAGCTATCGCTAAGCTACGGGAGGCGAATGATCGGTGCGGTCAGATAAATAGAGAAGGTTTCTTACATGACAAATCCTTGTCATGGGATCCGGATGTTTTACAAACCAGCATCCGTAGCCTTATTGGCAACCTCCTCTCTGGCTACCGATCGTCGTTGTTTGGGCAATGCACGTTCTCCAACGGTGCCTCTATGGGGCACAAGTTGCAGGATGCAGCGCCCTACAAGAAGTTCGCTGAACAAGCAACCGTTACCCCCCGCGCTCTGAGAGCGGCTCTATTGGTCCGAGACCAATGTGCGCCGTGGATCAGACACGCGGTCCGCTATAACGAGTCATATGAGTTTAGGCTCGTTGTAGGGAACGGAGTGTTTACAGTTCCGAAGAATAATAAAATAGATCGGGCTGCCTGTAAGGAGCCTGATATGAATATGTACCTCCAGAAAGGGGTCGGTGCCTTTATCAGACGCCGGCTCAAATCCGTTGGTATAGACCTGAATGATCAATCGATCAACCAGCGTCTGGCTCAGCAGGGCAGCGTAGATGGTTCGCTTGCGACGATAGACTTATCGTCTGCATCCGATTCCATCTCCGATCGCCTGGTGTGGAGTTTTCTCCCACCTGAGCTATATTCATATCTCGATCGTATCCGCTCACACTACGGAATCGTAGATGGCGAGACGATACGATGGGAACTATTTTCCACAATGGGAAATGGGTTCACATTTGAGCTAGAGTCCATGATATTCTGGGCAATAGTCAAAGCGACCCAAATCCATTTTGGTAACGCCGGAACCATAGGCATCTACGGGAGCGTTATTATATGCCCCAGTGAGATTGCACCCCGTGTGCTAGAGGCACTTGCCTACTACGGTTTTAAACCGAATCTTCGCAAAACGTTCGTGTCCGGGCTCTTTCGCGAGAGCTGCGGCGCGCACTTTTACCGTGGTGTCGATGTCAAACCGTTTTACATCAAGAAACCTGTTGACAATCTCTTCGCCCTGATGCTGATATTAAATCGGCTACGGGGTTGGGGAGTTGTCGGAGGTATGTCAGATCCACGCCTCTACAAGGTGTGGGTACGGCTCTCCTCCCAGGTGCCTTCGATGTTCTTCGGTGGGACGGACCTCGCTGCCGACTACTACGTAGTCAGCCCGCCTACGGCAGTCTCGGTATACACCAAGACTCCGTACGGGCGGCTGCTCGCGGATACCCGTACCTCGGGTTTCCGTCTTGCTCGTATCGCTCGAGAACGCAAGTTCTTCAGCGAAAAGCACGACAGTGGTCGCTACATAGCGTGGTTCCATACTGGAGGTGAAATCACCGACAGCATGAAGTCCGCCGGCGTGCGCGTTATACGCACTTCGGAGTGGCTAACGCCGGTTCCCACATTCCCTCAGGAGTGTGGGCCAGCGAGCTCTCCTCGGTAGctgaccgagggacccccgtaaacggggtgggtgtgctcgaaagagcacgggtccgcgaaagcggtggctccaccgaaaggtgggcgggcttcggcccagggacctccccctaaagagaggacccgggattctcccgatttggtaactagctgcttggctagttaccaccc** |

| **[*α*]_MS2_ (-) (T7 promoter-cUTR_T_ -antisense α-antisense RBS_2_-cUTR_L_)** |
| --- |
| **taatacgactcactatagggtggtaactagccaagcagctagttaccaaatcgggagaatcccgggtcctctctttagggggaggtccctgggccgaagcccgcccacctttcggtggagccggaccgctttcgcacccgtgctctttcgagcacacccaccccgtttacgggggtccctcggtcagctaccgaggagaaaacttaaCTAGCGCCATTCGCCATTCAGGCTGCGCAACTGTTGGGAAGGGCGATCGGTGCGGGCCTCTTCGCTATTACGCCAGCTGGCGAAAGGGGGATGTGCTGCAAGGCGATTAAGTTGGGTAACGCCAGGGTTTTCCCAGTCACGACGTTGTAAAACGACGGCCAGTGAATTCGTAATCATGGTCATagctgtttcctgtgtgaaattgttatccgctcacaattgttttcccgaaacctcctaggaatggaattccggctacctacagcgatagccatggtagcgtctcgctaaagacattaaaaatggcattagctcgacaggaagttgagcaggaccccgaaaggggtcccaccc** |
| **lacZΔM15 (+) (T7 promoter-RBS-His6-linker-lacZΔM15)** |
| **taatacgactcactataggggaattgtgagcggataacaattcccctctagaaataattttgtttaactttaagaaggagatataccATGGGCAGCAGCCATCATCATCATCATCACGGTGGATCTGGAGGTTCAGGTGGAAGTCCTAGGATGACCATGATTACGGATTCACTGGCCGTCGTGGCCCGCACCGATCGCCCTTCCCAACAGTTGCGCAGCCTGAATGGCGAATGGCGCTTTGCCTGGTTTCCGGCACCAGAAGCGGTGCCGGAAAGCTGGCTGGAGTGCGATCTTCCTGAGGCCGATACTGTCGTCGTCCCCTCAAACTGGCAGATGCACGGTTACGATGCGCCCATCTACACCAACGTGACCTATCCCATTACGGTCAATCCGCCGTTTGTTCCCACGGAGAATCCGACGGGTTGTTACTCGCTCACATTTAATGTTGATGAAAGCTGGCTACAGGAAGGCCAGACGCGAATTATTTTTGATGGCGTTAACTCGGCGTTTCATCTGTGGTGCAACGGGCGCTGGGTCGGTTACGGCCAGGACAGTCGTTTGCCGTCTGAATTTGACCTGAGCGCATTTTTACGCGCCGGAGAAAACCGCCTCGCGGTGATGGTGCTGCGCTGGAGTGACGGCAGTTATCTGGAAGATCAGGATATGTGGCGGATGAGCGGCATTTTCCGTGACGTCTCGTTGCTGCATAAACCGACTACACAAATCAGCGATTTCCATGTTGCCACTCGCTTTAATGATGATTTCAGCCGCGCTGTACTGGAGGCTGAAGTTCAGATGTGCGGCGAGTTGCGTGACTACCTACGGGTAACAGTTTCTTTATGGCAGGGTGAAACGCAGGTCGCCAGCGGCACCGCGCCTTTCGGCGGTGAAATTATCGATGAGCGTGGTGGTTATGCCGATCGCGTCACACTACGTCTGAACGTCGAAAACCCGAAACTGTGGAGCGCCGAAATCCCGAATCTCTATCGTGCGGTGGTTGAACTGCACACCGCCGACGGCACGCTGATTGAAGCAGAAGCCTGCGATGTCGGTTTCCGCGAGGTGCGGATTGAAAATGGTCTGCTGCTGCTGAACGGCAAGCCGTTGCTGATTCGAGGCGTTAACCGTCACGAGCATCATCCTCTGCATGGTCAGGTCATGGATGAGCAGACGATGGTGCAGGATATCCTGCTGATGAAGCAGAACAACTTTAACGCCGTGCGCTGTTCGCATTATCCGAACCATCCGCTGTGGTACACGCTGTGCGACCGCTACGGCCTGTATGTGGTGGATGAAGCCAATATTGAAACCCACGGCATGGTGCCAATGAATCGTCTGACCGATGATCCGCGCTGGCTACCGGCGATGAGCGAACGCGTAACGCGAATGGTGCAGCGCGATCGTAATCACCCGAGTGTGATCATCTGGTCGCTGGGGAATGAATCAGGCCACGGCGCTAATCACGACGCGCTGTATCGCTGGATCAAATCTGTCGATCCTTCCCGCCCGGTGCAGTATGAAGGCGGCGGAGCCGACACCACGGCCACCGATATTATTTGCCCGATGTACGCGCGCGTGGATGAAGACCAGCCCTTCCCGGCTGTGCCGAAATGGTCCATCAAAAAATGGCTTTCGCTACCTGGAGAGACGCGCCCGCTGATCCTTTGCGAATACGCCCACGCGATGGGTAACAGTCTTGGCGGTTTCGCTAAATACTGGCAGGCGTTTCGTCAGTATCCCCGTTTACAGGGCGGCTTCGTCTGGGACTGGGTGGATCAGTCGCTGATTAAATATGATGAAAACGGCAACCCGTGGTCGGCTTACGGCGGTGATTTTGGCGATACGCCGAACGATCGCCAGTTCTGTATGAACGGTCTGGTCTTTGCCGACCGCACGCCGCATCCAGCGCTGACGGAAGCAAAACACCAGCAGCAGTTTTTCCAGTTCCGTTTATCCGGGCAAACCATCGAAGTGACCAGCGAATACCTGTTCCGTCATAGCGATAACGAGCTCCTGCACTGGATGGTGGCGCTGGATGGTAAGCCGCTGGCAAGCGGTGAAGTGCCTCTGGATGTCGCTCCACAAGGTAAACAGTTGATTGAACTGCCTGAACTACCGCAGCCGGAGAGCGCCGGGCAACTCTGGCTCACAGTACGCGTAGTGCAACCGAACGCGACCGCATGGTCAGAAGCCGGGCACATCAGCGCCTGGCAGCAGTGGCGTCTGGCGGAAAACCTCAGTGTGACGCTCCCCGCCGCGTCCCACGCCATCCCGCATCTGACCACCAGCGAAATGGATTTTTGCATCGAGCTGGGTAATAAGCGTTGGCAATTTAACCGCCAGTCAGGCTTTCTTTCACAGATGTGGATTGGCGATAAAAAACAACTGCTGACGCCGCTGCGCGATCAGTTCACCCGTGCACCGCTGGATAACGACATTGGCGTAAGTGAAGCGACCCGCATTGACCCTAACGCCTGGGTCGAACGCTGGAAGGCGGCGGGCCATTACCAGGCCGAAGCAGCGTTGTTGCAGTGCACGGCAGATACACTTGCTGATGCGGTGCTGATTACGACCGCTCACGCGTGGCAGCATCAGGGGAAAACCTTATTTATCAGCCGGAAAACCTACCGGATTGATGGTAGTGGTCAAATGGCGATTACCGTTGATGTTGAAGTGGCGAGCGATACACCGCATCCGGCGCGGATTGGCCTGAACTGCCAGCTGGCGCAGGTAGCAGAGCGGGTAAACTGGCTCGGATTAGGGCCGCAAGAAAACTATCCCGACCGCCTTACTGCCGCCTGTTTTGACCGCTGGGATCTGCCATTGTCAGACATGTATACCCCGTACGTCTTCCCGAGCGAAAACGGTCTGCGCTGCGGGACGCGCGAATTGAATTATGGCCCACACCAGTGGCGCGGCGACTTCCAGTTCAACATCAGCCGCTACAGTCAACAGCAACTGATGGAAACCAGCCATCGCCATCTGCTGCACGCGGAAGAAGGCACATGGCTGAATATCGACGGTTTCCATATGGGGATTGGTGGCGACGACTCCTGGAGCCCGTCAGTATCGGCGGAATTCCAGCTGAGCGCCGGTCGCTACCATTACCAGTTGGTCTGGTGTCAAAAATAA** |
| **[*α*]_minMS2_ (-) (T7 promoter-minimised cUTR_T_ -antisense α-antisense RBS_2_-minimised cUTR_L_)** |
| **taatacgactcactatagggtggtaactagccaagcagctagttaccaaatcgggagaatcccgggtcctctctttagggggaggtccctgggc**  **aaaacttaaCTAGCGCCATTCGCCATTCAGGCTGCGCAACTGTTGGGAAGGGCGATCGGTGCGGGCCTCTTCGCTATTACGCCAGCTGGCGAAAGGGGGATGTGCTGCAAGGCGATTAAGTTGGGTAACGCCAGGGTTTTCCCAGTCACGACGTTGTAAAACGACGGCCAGTGAATTCGTAATCATGGTCATagctgtttcctgtgtgaaattgttatccgctcacaattgttttcccggctcgacaggaagttgagcaggaccccgaaaggggtcccaccc** |
| **[F30-Bro]_MS2_ (-) (T7 promoter-cUTR_T_ -antisense F30-Bro-cUTR_L_)** |
| **taatacgactcactatagggtggtaactagccaagcagctagttaccaaatcgggagaatcccgggtcctctctttagggggaggtccctgggccgaagcccgcccacctttcggtggagccggaccgctttcgcacccgtgctctttcgagcacacccaccccgtttacgggggtccctcggtcagctaccgaggagTTGCCATGAATGATCCCGAAGGATCATCAGAGTATGTGGGAGCCCACACTCTACTCGACAGATACGAATATCTGGACCCGACCGTCTCCCACATACACATGGCAAaaacctcctaggaatggaattccggctacctacagcgatagccatggtagcgtctcgctaaagacattaaaaatggcattagctcgacaggaagttgagcaggaccccgaaaggggtcccaccc** |
| **[*sfGFP*]_MS2_ (-) (T7 promoter-cUTR_T_ -antisense sfGFP-antisense RBS_1_-cUTR_L_)** |
| **taatacgactcactatagggtggtaactagccaagcagctagttaccaaatcgggagaatcccgggtcctctctttagggggaggtccctgggccgaagcccgcccacctttcggtggagccggaccgctttcgcacccgtgctctttcgagcacacccaccccgtttacgggggtccctcggtcagctaccgaggagaaaacttaaCTATTTGTAGAGCTCATCCATGCCATGTGTAATCCCAGCAGCAGTTACAAACTCAAGAAGGACCATGTGGTCACGCTTTTCGTTGGGATCTTTCGAAAGGACAGATTGTGTCGACAGGTAATGGTTGTCTGGTAAAAGGACAGGGCCATCGCCAATTGGAGTATTTTGTTGATAATGGTCTGCTAGTTGAACGGAACCATCTTCAACGTTGTGGCGAATTTTGAAGTTAGCTTTGATTCCATTCTTTTGTTTGTCTGCCGTGATGTATACATTGTGTGAGTTAAAGTTGTACTCGAGTTTGTGTCCGAGAATGTTTCCATCTTCTTTAAAATCAATACCTTTTAACTCGATACGATTAACAAGGGTATCACCTTCAAACTTGACTTCAGCACGCGTCTTGTAGGTCCCGTCATCTTTGAAAGATATAGTGCGTTCCTGTACATAACCTTCGGGCATGGCACTCTTGAAAAAGTCATGCCGTTTCATGTGATCCGGATAACGGGAAAAGCATTGAACACCATAGGTCAGAGTAGTGACAAGTGTTGGCCATGGAACAGGTAGTTTTCCAGTAGTGCAAATAAATTTAAGGGTGAGTTTTCCGTTTGTAGCATCACCTTCACCCTCTCCACGGACAGAAAATTTGTGCCCATTAACATCACCATCTAATTCAACAAGAATTGGGACAACTCCAGTGAAAAGTTCTTCTCCTTTGCTCATattttttcctccttatacttaagcccaaacctcctaggaatggaattccggctacctacagcgatagccatggtagcgtctcgctaaagacattaaaaatggcattagctcgacaggaagttgagcaggaccccgaaaggggtcccaccc** |
| **[*lacZ*]_MS2_ (-) (T7 promoter-cUTR_T_ -antisense lacZ-antisense RBS_1_-cUTR_L_)** |
| **taatacgactcactatagggtggtaactagccaagcagctagttaccaaatcgggagaatcccgggtcctctctttagggggaggtccctgggccgaagcccgcccacctttcggtggagccggaccgctttcgcacccgtgctctttcgagcacacccaccccgtttacgggggtccctcggtcagctaccgaggagaaaacttaaTTATTTTTGACACCAGACCAACTGGTAATGGTAGCGACCGGCGCTCAGCTGGAATTCCGCCGATACTGACGGGCTCCAGGAGTCGTCGCCACCAATCCCCATATGGAAACCGTCGATATTCAGCCATGTGCCTTCTTCCGCGTGCAGCAGATGGCGATGGCTGGTTTCCATCAGTTGCTGTTGACTGTAGCGGCTGATGTTGAACTGGAAGTCGCCGCGCCACTGGTGTGGGCCATAATTCAATTCGCGCGTCCCGCAGCGCAGACCGTTTTCGCTCGGGAAGACGTACGGGGTATACATGTCTGACAATGGCAGATCCCAGCGGTCAAAACAGGCGGCAGTAAGGCGGTCGGGATAGTTTTCTTGCGGCCCTAATCCGAGCCAGTTTACCCGCTCTGCTACCTGCGCCAGCTGGCAGTTCAGGCCAATCCGCGCCGGATGCGGTGTATCGCTCGCCACTTCAACATCAACGGTAATCGCCATTTGACCACTACCATCAATCCGGTAGGTTTTCCGGCTGATAAATAAGGTTTTCCCCTGATGCTGCCACGCGTGAGCGGTCGTAATCAGCACCGCATCAGCAAGTGTATCTGCCGTGCACTGCAACAACGCTGCTTCGGCCTGGTAATGGCCCGCCGCCTTCCAGCGTTCGACCCAGGCGTTAGGGTCAATGCGGGTCGCTTCACTTACGCCAATGTCGTTATCCAGCGGTGCACGGGTGAACTGATCGCGCAGCGGCGTCAGCAGTTGTTTTTTATCGCCAATCCACATCTGTGAAAGAAAGCCTGACTGGCGGTTAAATTGCCAACGCTTATTACCCAGCTCGATGCAAAAATCCATTTCGCTGGTGGTCAGATGCGGGATGGCGTGGGACGCGGCGGGGAGCGTCACACTGAGGTTTTCCGCCAGACGCCACTGCTGCCAGGCGCTGATGTGCCCGGCTTCTGACCATGCGGTCGCGTTCGGTTGCACTACGCGTACTGTGAGCCAGAGTTGCCCGGCGCTCTCCGGCTGCGGTAGTTCAGGCAGTTCAATCAACTGTTTACCTTGTGGAGCGACATCCAGAGGCACTTCACCGCTTGCCAGCGGCTTACCATCCAGCGCCACCATCCAGTGCAGGAGCTCGTTATCGCTATGACGGAACAGGTATTCGCTGGTCACTTCGATGGTTTGCCCGGATAAACGGAACTGGAAAAACTGCTGCTGGTGTTTTGCTTCCGTCAGCGCTGGATGCGGCGTGCGGTCGGCAAAGACCAGACCGTTCATACAGAACTGGCGATCGTTCGGCGTATCGCCAAAATCACCGCCGTAAGCCGACCACGGGTTGCCGTTTTCATCATATTTAATCAGCGACTGATCCACCCAGTCCCAGACGAAGCCGCCCTGTAAACGGGGATACTGACGAAACGCCTGCCAGTATTTAGCGAAACCGCCAAGACTGTTACCCATCGCGTGGGCGTATTCGCAAAGGATCAGCGGGCGCGTCTCTCCAGGTAGCGAAAGCCATTTTTTGATGGACCATTTCGGCACAGCCGGGAAGGGCTGGTCTTCATCCACGCGCGCGTACATCGGGCAAATAATATCGGTGGCCGTGGTGTCGGCTCCGCCGCCTTCATACTGCACCGGGCGGGAAGGATCGACAGATTTGATCCAGCGATACAGCGCGTCGTGATTAGCGCCGTGGCCTGATTCATTCCCCAGCGACCAGATGATCACACTCGGGTGATTACGATCGCGCTGCACCATTCGCGTTACGCGTTCGCTCATCGCCGGTAGCCAGCGCGGATCATCGGTCAGACGATTCATTGGCACCATGCCGTGGGTTTCAATATTGGCTTCATCCACCACATACAGGCCGTAGCGGTCGCACAGCGTGTACCACAGCGGATGGTTCGGATAATGCGAACAGCGCACGGCGTTAAAGTTGTTCTGCTTCATCAGCAGGATATCCTGCACCATCGTCTGCTCATCCATGACCTGACCATGCAGAGGATGATGCTCGTGACGGTTAACGCCTCGAATCAGCAACGGCTTGCCGTTCAGCAGCAGCAGACCATTTTCAATCCGCACCTCGCGGAAACCGACATCGCAGGCTTCTGCTTCAATCAGCGTGCCGTCGGCGGTGTGCAGTTCAACCACCGCACGATAGAGATTCGGGATTTCGGCGCTCCACAGTTTCGGGTTTTCGACGTTCAGACGTAGTGTGACGCGATCGGCATAACCACCACGCTCATCGATAATTTCACCGCCGAAAGGCGCGGTGCCGCTGGCGACCTGCGTTTCACCCTGCCATAAAGAAACTGTTACCCGTAGGTAGTCACGCAACTCGCCGCACATCTGAACTTCAGCCTCCAGTACAGCGCGGCTGAAATCATCATTAAAGCGAGTGGCAACATGGAAATCGCTGATTTGTGTAGTCGGTTTATGCAGCAACGAGACGTCACGGAAAATGCCGCTCATCCGCCACATATCCTGATCTTCCAGATAACTGCCGTCACTCCAGCGCAGCACCATCACCGCGAGGCGGTTTTCTCCGGCGCGTAAAAATGCGCTCAGGTCAAATTCAGACGGCAAACGACTGTCCTGGCCGTAACCGACCCAGCGCCCGTTGCACCACAGATGAAACGCCGAGTTAACGCCATCAAAAATAATTCGCGTCTGGCCTTCCTGTAGCCAGCTTTCATCAACATTAAATGTGAGCGAGTAACAACCCGTCGGATTCTCCGTGGGAACAAACGGCGGATTGACCGTAATGGGATAGGTCACGTTGGTGTAGATGGGCGCATCGTAACCGTGCATCTGCCAGTTTGAGGGGACGACGACAGTATCGGCCTCAGGAAGATCGCACTCCAGCCAGCTTTCCGGCACCGCTTCTGGTGCCGGAAACCAGGCAAAGCGCCATTCGCCATTCAGGCTGCGCAACTGTTGGGAAGGGCGATCGGTGCGGGCCTCTTCGCTATTACGCCAGCTGGCGAAAGGGGGATGTGCTGCAAGGCGATTAAGTTGGGTAACGCCAGGGTTTTCCCAGTCACGACGTTGTAAAACGACGGGATCGATCTCGCCATACAGCGCGTTGAAACGCTGGGCAATATCGCGGCTCAGTTCGAGGTGCTGTTTCTGGTCTTCACCCACCGGTACCAGACCGCCACGGCTTACGGCAATAATGCCTTTCCATTGTTCAGAAGGCATCAGTCGGCTTGCGAGTTTACGTGCATGGATCTGCAACATGTCCCAGGTGACGATGTATTTTTCGCTCATattttttcctccttatacttaagcccaaacctcctaggaatggaattccggctacctacagcgatagccatggtagcgtctcgctaaagacattaaaaatggcattagctcgacaggaagttgagcaggaccccgaaaggggtcccaccc** |
| **Shortened [F30-Bro]_MS2_ (-) (T7 promoter-antisense F30-Bro-cUTR_L_)** |
| **taatacgactcactatagggTTGCCATGAATGATCCCGAAGGATCATCAGAGTATGTGGGAGCCCACACTCTACTCGACAGATACGAATATCTGGACCCGACCGTCTCCCACATACACATGGCAAaaacctcctaggaatggaattccggctacctacagcgatagccatggtagcgtctcgctaaagacattaaaaatggcattagctcgacaggaagttgagcaggaccccgaaaggggtcccaccc** |
| **Shortened [*sfGFP*-RBS_2_]_MS2_ (-) (T7 promoter-antisense sfGFP-antisense RBS_2_-cUTR_L_)** |
| **taatacgactcactatagggagaaaacttaaCTATTTGTAGAGCTCATCCATGCCATGTGTAATCCCAGCAGCAGTTACAAACTCAAGAAGGACCATGTGGTCACGCTTTTCGTTGGGATCTTTCGAAAGGACAGATTGTGTCGACAGGTAATGGTTGTCTGGTAAAAGGACAGGGCCATCGCCAATTGGAGTATTTTGTTGATAATGGTCTGCTAGTTGAACGGAACCATCTTCAACGTTGTGGCGAATTTTGAAGTTAGCTTTGATTCCATTCTTTTGTTTGTCTGCCGTGATGTATACATTGTGTGAGTTAAAGTTGTACTCGAGTTTGTGTCCGAGAATGTTTCCATCTTCTTTAAAATCAATACCTTTTAACTCGATACGATTAACAAGGGTATCACCTTCAAACTTGACTTCAGCACGCGTCTTGTAGGTCCCGTCATCTTTGAAAGATATAGTGCGTTCCTGTACATAACCTTCGGGCATGGCACTCTTGAAAAAGTCATGCCGTTTCATGTGATCCGGATAACGGGAAAAGCATTGAACACCATAGGTCAGAGTAGTGACAAGTGTTGGCCATGGAACAGGTAGTTTTCCAGTAGTGCAAATAAATTTAAGGGTGAGTTTTCCGTTTGTAGCATCACCTTCACCCTCTCCACGGACAGAAAATTTGTGCCCATTAACATCACCATCTAATTCAACAAGAATTGGGACAACTCCAGTGAAAAGTTCTTCTCCTTTGCTCATagctgtttcctgtgtgaaattgttatccgctcacaattgttttcccgaaacctcctaggaatggaattccggctacctacagcgatagccatggtagcgtctcgctaaagacattaaaaatggcattagctcgacaggaagttgagcaggaccccgaaaggggtcccaccc** |
| **[*SP6 pol*]_MS2_ (-) (T7 promoter-cUTR_T_ -antisense SP6 pol-antisense RBS_1_-cUTR_L_)** |
| **taatacgactcactatagggtggtaactagccaagcagctagttaccaaatcgggagaatcccgggtcctctctttagggggaggtccctgggccgaagcccgcccacctttcggtggagccggaccgctttcgcacccgtgctctttcgagcacacccaccccgtttacgggggtccctcggtcagctaccgaggagaaaacttaaTTAGGCAAATACGTATTCAGAATCCATGATTTCGTTAAGGTCGAACTCCCCTTGCTCAGGTACTTCGATACCTGTATCAACCATCCAGCGCTCTTCATGCTCCTCCAGTAGTTTCTGAAGCGCATTACCATCAATATACATTGCAACCATCTGCCCTTTAAGTGCCACTCTAAGAGTGAGGGTGTTGTCTGCATGAGTACCAAAAGAGTCGTGGATTACAGCGATACTAGTTACGCCCTTGTCTACCAATTCACATACGGTAAGGATAAGGTGACTTGCGTCATGACCGTGTACGAAATTAGGTGCTGCTGCTCCCATCATAGCGGCTTCATCTACGATATCCGTTTCAACCTGAAGGGACATCTTGATATCACCCATCAGACAGGTACGCACGCGTAGCATCTCGGTTGCCATGATCTTCTGTTCTAAGATGAAGCCAGTAGGCAGGGTGTACATCAGGCCTTCATTACGTTTCGCTGCAAAGCGTGCAAGCTGGCGTATCATCTTCATAGCTACTATCGGTGCCTTAACTACTTCAGAAATAGAAGGCCAGATTAGTGCCGTCATGTAGTTGTAAGCTGCGCCCGGAGTCAAGTAATCTTGACGATCGTCTTCAAAAGGATGTACCTTGTTTGCCGTCCGCCCTTCTGCTACTGCCTTCTGCGCCTCTTTTTCCTCTAAGTCTACGATGTAATCAATCACAGATTCACGGCAAGTTAAGCGAGTAGAACCATATGGCAAGGTCATCACGGGCTTTTTGGTTAAGCTACGGGTAATACCAATACTATCCCATGCGCTAGCCATTGCTCGCAGTTCTGTACCGGACAGCGTGACGCTACCAGAAGTAAACGTGGTTGCATCGTCCGCATCCATATATAGCGCATTCTTCTTGATAACCACTTGCGCCACCGCCCCATAGATATCCTGCGGTGCATCGGAGGGTTTCAGGTTAACAGCTTTGGCCCCTACTTCGTCGCGAAGCATAGCACTATAGTGCTGAATGCCTGAACAAGACCCGTCCTGATGTACTGGTAGGTGAGTGCGGAATTCGTCGGCCCTTCCTTCATCCACCAAATCAAGGTATTGAGCATACTCAAAGCACCAAGCGAGGAATTCATAAGGTGCATCAGCTTTAGCCCATTGGGTGAATGTGAGAGGGTCTGCGGCGATGTCTCGACACATATCTTGGAATTCCTCATCTAATACGTTAGACACGCGCACATCAAAAGTTTTCTTGTCCCATCCCCAAAGGTTAGCACCATTGATGCAGAACCATTTAAGCGCTTCTACGCCATTCACAGGGCGTCCCTCGGTAAAGCGGAGTAATGCCTTACCTAAGTCGTTAGACTGCGGAGAGAGCGTGCTAGATTGCACATAGACACGGCTGCGGCTATCCATTGCGTACACGAAGTAAATGGATTCAAAGGCGCTATATTTACGGGCCTGTCCTACCATGCGAACAACGGCGGCGGACTTTGAACCGCGCTTAGTTTCTGCGGTATATAGGCGCGCGCATTCGCCTTTCCAGTTAATGAATTGTTGCCACTGCTCAGGTGATAGCATCTCTTTCAGTTCACGACCGCGCAGGTGTTGGAATTCAACAGGTACCGGGTTAGCTGGCTTGTTCTCCTTGTCAATCAGTGGCTTGAAGGAAGGTACACCATAACCAAGGTCTAAGCGGATTACTTCTTCAATAACTGCTAATACATCCTTGTTGATTTGCCATTGTGTATTTTGTAATGCGTTGATAGCCTTATAAACCTTTGGCATTTGCTTTTGAGTCAACTTGCGTACATGCTCACGGTTACCTTTTACAAGACGGATACGGCTAGCTACCTTCTCAGTATGGAACCCTCCATTAAATGGAGTTCTCCAAGGACGAGGAGGGATTACGCAAGGGGCATAAGCTGGGCTTAATTGCGCTACGTGCTCTTTGAATGCGCTAATCCACTGGCCTACACTTTCAGAAGTTTGTAAGTAGTAAATAGTCTTTCCGCCATAAGTGCGCATAGCACGCATAAATACAGGTTCACCATTATAGAAAACGCTACCTTCTAAGATTTCAAGCAAGGTAGTACCAATCTGCAATTGAGTTTCTTTTGGCCACGCCTCCCAACGGTCAAAGTCCGCGTCCTTTTCTGCAACTGATTTTTCAGCAACTACAGCTACGTTATGAGCGTGACGATATGACTTAGTACGGCTAGCCTTGAGTGACTTCTTAACCTTCTCAAAGTATTTAGCGGCGTGACCTTCTAGCTTAGAAAAGCGCACTTGGTCTTCAATGCGTTCTGCTACACTCATTGCAATAGCCTGAAGGGTAGCATCCGTATTCAGCATATCCATAACAACTTTCATAGTGATGTATGCTGCAACTTCATTTTCTACACATTGTAAGAAAGCCAATGCGCGAGGTGCACGACCTTTCTTACCTTCGTACTCTTCTTTATAAGCCTGAATGCCTTCAGCCATAGGTGCAATAAGTTCTGACAACAGGCGGCGGTTCCATGCTGTGTCGCTCTCGCTACCTGCTGCAATCTGGCGTTGTTGATCTGCTTCGAAGCGACGAATGCCACCATTAAACATCTCTTCTTCTAATTGAAGCTGGATAGCGTGTAAATCTTGCATattttttcctccttatacttaagcccaaacctcctaggaatggaattccggctacctacagcgatagccatggtagcgtctcgctaaagacattaaaaatggcattagctcgacaggaagttgagcaggaccccgaaaggggtcccaccc** |
| **SP6-sfGFP DNA (+) (SP6 promoter-RBS_1_-sfGFP-UTR_T_)** |
| **atttaggtgacactatagaagggcttaagtataaggaggaaaaaatATGAGCAAAGGAGAAGAACTTTTCACTGGAGTTGTCCCAATTCTTGTTGAATTAGATGGTGATGTTAATGGGCACAAATTTTCTGTCCGTGGAGAGGGTGAAGGTGATGCTACAAACGGAAAACTCACCCTTAAATTTATTTGCACTACTGGAAAACTACCTGTTCCATGGCCAACACTTGTCACTACTCTGACCTATGGTGTTCAATGCTTTTCCCGTTATCCGGATCACATGAAACGGCATGACTTTTTCAAGAGTGCCATGCCCGAAGGTTATGTACAGGAACGCACTATATCTTTCAAAGATGACGGGACCTACAAGACGCGTGCTGAAGTCAAGTTTGAAGGTGATACCCTTGTTAATCGTATCGAGTTAAAAGGTATTGATTTTAAAGAAGATGGAAACATTCTCGGACACAAACTCGAGTACAACTTTAACTCACACAATGTATACATCACGGCAGACAAACAAAAGAATGGAATCAAAGCTAACTTCAAAATTCGCCACAACGTTGAAGATGGTTCCGTTCAACTAGCAGACCATTATCAACAAAATACTCCAATTGGCGATGGCCCTGTCCTTTTACCAGACAACCATTACCTGTCGACACAATCTGTCCTTTCGAAAGATCCCAACGAAAAGCGTGACCACATGGTCCTTCTTGAGTTTGTAACTGCTGCTGGGATTACACATGGCATGGATGAGCTCTACAAATAGTTAAGTTTTctcctcggtagctgaccgagggacccccgtaaacggggtgggtgtgctcgaaagagcacgggtgcgaaagcggtccggctccaccgaaaggtgggcgggcttcggcccagggacctccccctaaagagaggacccgggattctcccgatttggtaactagctgcttggctagttaccaccc** |
| **[*sfGFP*-RBS_2_]_MS2_ (-) (T7 promoter-cUTR_T_ -antisense sfGFP-antisense RBS_2_-cUTR_L_)** |
| **taatacgactcactatagggtggtaactagccaagcagctagttaccaaatcgggagaatcccgggtcctctctttagggggaggtccctgggccgaagcccgcccacctttcggtggagccggaccgctttcgcacccgtgctctttcgagcacacccaccccgtttacgggggtccctcggtcagctaccgaggagaaaacttaaCTATTTGTAGAGCTCATCCATGCCATGTGTAATCCCAGCAGCAGTTACAAACTCAAGAAGGACCATGTGGTCACGCTTTTCGTTGGGATCTTTCGAAAGGACAGATTGTGTCGACAGGTAATGGTTGTCTGGTAAAAGGACAGGGCCATCGCCAATTGGAGTATTTTGTTGATAATGGTCTGCTAGTTGAACGGAACCATCTTCAACGTTGTGGCGAATTTTGAAGTTAGCTTTGATTCCATTCTTTTGTTTGTCTGCCGTGATGTATACATTGTGTGAGTTAAAGTTGTACTCGAGTTTGTGTCCGAGAATGTTTCCATCTTCTTTAAAATCAATACCTTTTAACTCGATACGATTAACAAGGGTATCACCTTCAAACTTGACTTCAGCACGCGTCTTGTAGGTCCCGTCATCTTTGAAAGATATAGTGCGTTCCTGTACATAACCTTCGGGCATGGCACTCTTGAAAAAGTCATGCCGTTTCATGTGATCCGGATAACGGGAAAAGCATTGAACACCATAGGTCAGAGTAGTGACAAGTGTTGGCCATGGAACAGGTAGTTTTCCAGTAGTGCAAATAAATTTAAGGGTGAGTTTTCCGTTTGTAGCATCACCTTCACCCTCTCCACGGACAGAAAATTTGTGCCCATTAACATCACCATCTAATTCAACAAGAATTGGGACAACTCCAGTGAAAAGTTCTTCTCCTTTGCTCATagctgtttcctgtgtgaaattgttatccgctcacaattgttttcccgaaacctcctaggaatggaattccggctacctacagcgatagccatggtagcgtctcgctaaagacattaaaaatggcattagctcgacaggaagttgagcaggaccccgaaaggggtcccaccc** |
| **[*lacZ*-RBS_2_]_MS2_ (-) (T7 promoter-cUTR_T_ -antisense lacZ-antisense RBS_2_-cUTR_L_)** |
| **taatacgactcactatagggtggtaactagccaagcagctagttaccaaatcgggagaatcccgggtcctctctttagggggaggtccctgggccgaagcccgcccacctttcggtggagccggaccgctttcgcacccgtgctctttcgagcacacccaccccgtttacgggggtccctcggtcagctaccgaggagaaaacttaaTTATTTTTGACACCAGACCAACTGGTAATGGTAGCGACCGGCGCTCAGCTGGAATTCCGCCGATACTGACGGGCTCCAGGAGTCGTCGCCACCAATCCCCATATGGAAACCGTCGATATTCAGCCATGTGCCTTCTTCCGCGTGCAGCAGATGGCGATGGCTGGTTTCCATCAGTTGCTGTTGACTGTAGCGGCTGATGTTGAACTGGAAGTCGCCGCGCCACTGGTGTGGGCCATAATTCAATTCGCGCGTCCCGCAGCGCAGACCGTTTTCGCTCGGGAAGACGTACGGGGTATACATGTCTGACAATGGCAGATCCCAGCGGTCAAAACAGGCGGCAGTAAGGCGGTCGGGATAGTTTTCTTGCGGCCCTAATCCGAGCCAGTTTACCCGCTCTGCTACCTGCGCCAGCTGGCAGTTCAGGCCAATCCGCGCCGGATGCGGTGTATCGCTCGCCACTTCAACATCAACGGTAATCGCCATTTGACCACTACCATCAATCCGGTAGGTTTTCCGGCTGATAAATAAGGTTTTCCCCTGATGCTGCCACGCGTGAGCGGTCGTAATCAGCACCGCATCAGCAAGTGTATCTGCCGTGCACTGCAACAACGCTGCTTCGGCCTGGTAATGGCCCGCCGCCTTCCAGCGTTCGACCCAGGCGTTAGGGTCAATGCGGGTCGCTTCACTTACGCCAATGTCGTTATCCAGCGGTGCACGGGTGAACTGATCGCGCAGCGGCGTCAGCAGTTGTTTTTTATCGCCAATCCACATCTGTGAAAGAAAGCCTGACTGGCGGTTAAATTGCCAACGCTTATTACCCAGCTCGATGCAAAAATCCATTTCGCTGGTGGTCAGATGCGGGATGGCGTGGGACGCGGCGGGGAGCGTCACACTGAGGTTTTCCGCCAGACGCCACTGCTGCCAGGCGCTGATGTGCCCGGCTTCTGACCATGCGGTCGCGTTCGGTTGCACTACGCGTACTGTGAGCCAGAGTTGCCCGGCGCTCTCCGGCTGCGGTAGTTCAGGCAGTTCAATCAACTGTTTACCTTGTGGAGCGACATCCAGAGGCACTTCACCGCTTGCCAGCGGCTTACCATCCAGCGCCACCATCCAGTGCAGGAGCTCGTTATCGCTATGACGGAACAGGTATTCGCTGGTCACTTCGATGGTTTGCCCGGATAAACGGAACTGGAAAAACTGCTGCTGGTGTTTTGCTTCCGTCAGCGCTGGATGCGGCGTGCGGTCGGCAAAGACCAGACCGTTCATACAGAACTGGCGATCGTTCGGCGTATCGCCAAAATCACCGCCGTAAGCCGACCACGGGTTGCCGTTTTCATCATATTTAATCAGCGACTGATCCACCCAGTCCCAGACGAAGCCGCCCTGTAAACGGGGATACTGACGAAACGCCTGCCAGTATTTAGCGAAACCGCCAAGACTGTTACCCATCGCGTGGGCGTATTCGCAAAGGATCAGCGGGCGCGTCTCTCCAGGTAGCGAAAGCCATTTTTTGATGGACCATTTCGGCACAGCCGGGAAGGGCTGGTCTTCATCCACGCGCGCGTACATCGGGCAAATAATATCGGTGGCCGTGGTGTCGGCTCCGCCGCCTTCATACTGCACCGGGCGGGAAGGATCGACAGATTTGATCCAGCGATACAGCGCGTCGTGATTAGCGCCGTGGCCTGATTCATTCCCCAGCGACCAGATGATCACACTCGGGTGATTACGATCGCGCTGCACCATTCGCGTTACGCGTTCGCTCATCGCCGGTAGCCAGCGCGGATCATCGGTCAGACGATTCATTGGCACCATGCCGTGGGTTTCAATATTGGCTTCATCCACCACATACAGGCCGTAGCGGTCGCACAGCGTGTACCACAGCGGATGGTTCGGATAATGCGAACAGCGCACGGCGTTAAAGTTGTTCTGCTTCATCAGCAGGATATCCTGCACCATCGTCTGCTCATCCATGACCTGACCATGCAGAGGATGATGCTCGTGACGGTTAACGCCTCGAATCAGCAACGGCTTGCCGTTCAGCAGCAGCAGACCATTTTCAATCCGCACCTCGCGGAAACCGACATCGCAGGCTTCTGCTTCAATCAGCGTGCCGTCGGCGGTGTGCAGTTCAACCACCGCACGATAGAGATTCGGGATTTCGGCGCTCCACAGTTTCGGGTTTTCGACGTTCAGACGTAGTGTGACGCGATCGGCATAACCACCACGCTCATCGATAATTTCACCGCCGAAAGGCGCGGTGCCGCTGGCGACCTGCGTTTCACCCTGCCATAAAGAAACTGTTACCCGTAGGTAGTCACGCAACTCGCCGCACATCTGAACTTCAGCCTCCAGTACAGCGCGGCTGAAATCATCATTAAAGCGAGTGGCAACATGGAAATCGCTGATTTGTGTAGTCGGTTTATGCAGCAACGAGACGTCACGGAAAATGCCGCTCATCCGCCACATATCCTGATCTTCCAGATAACTGCCGTCACTCCAGCGCAGCACCATCACCGCGAGGCGGTTTTCTCCGGCGCGTAAAAATGCGCTCAGGTCAAATTCAGACGGCAAACGACTGTCCTGGCCGTAACCGACCCAGCGCCCGTTGCACCACAGATGAAACGCCGAGTTAACGCCATCAAAAATAATTCGCGTCTGGCCTTCCTGTAGCCAGCTTTCATCAACATTAAATGTGAGCGAGTAACAACCCGTCGGATTCTCCGTGGGAACAAACGGCGGATTGACCGTAATGGGATAGGTCACGTTGGTGTAGATGGGCGCATCGTAACCGTGCATCTGCCAGTTTGAGGGGACGACGACAGTATCGGCCTCAGGAAGATCGCACTCCAGCCAGCTTTCCGGCACCGCTTCTGGTGCCGGAAACCAGGCAAAGCGCCATTCGCCATTCAGGCTGCGCAACTGTTGGGAAGGGCGATCGGTGCGGGCCTCTTCGCTATTACGCCAGCTGGCGAAAGGGGGATGTGCTGCAAGGCGATTAAGTTGGGTAACGCCAGGGTTTTCCCAGTCACGACGTTGTAAAACGACGGGATCGATCTCGCCATACAGCGCGTTGAAACGCTGGGCAATATCGCGGCTCAGTTCGAGGTGCTGTTTCTGGTCTTCACCCACCGGTACCAGACCGCCACGGCTTACGGCAATAATGCCTTTCCATTGTTCAGAAGGCATCAGTCGGCTTGCGAGTTTACGTGCATGGATCTGCAACATGTCCCAGGTGACGATGTATTTTTCGCTCATagctgtttcctgtgtgaaattgttatccgctcacaattgttttcccgaaacctcctaggaatggaattccggctacctacagcgatagccatggtagcgtctcgctaaagacattaaaaatggcattagctcgacaggaagttgagcaggaccccgaaaggggtcccaccc** |
| **[F30-Bro]_MS2_ (+) (T7 promoter-UTR_L_-F30-Bro-UTR_T_)** |
| **gaaataatacgactcactatagggtgggacccctttcggggtcctgctcaacttcctgtcgagctaatgccatttttaatgtctttagcgagacgctaccatggctatcgctgtaggtagccggaattccattcctaggaggtttTTGCCATGTGTATGTGGGAGACGGTCGGGTCCAGATATTCGTATCTGTCGAGTAGAGTGTGGGCTCCCACATACTCTGATGATCCTTCGGGATCATTCATGGCAActcctcggtagctgaccgagggacccccgtaaacggggtgggtgtgctcgaaagagcacgggtgcgaaagcggtccggctccaccgaaaggtgggcgggcttcggcccagggacctccccctaaagagaggacccgggattctcccgatttggtaactagctgcttggctagttaccaccc** |

**Supplementary References**

1. Dai,X., Li,Z., Lai,M., Shu,S., Du,Y., Zhou,Z.H. and Sun,R. (2017) In situ structures of the genome and genome-delivery apparatus in a single-stranded RNA virus. *Nature*, **541**, 112–116.

2. Lorenz,R., Bernhart,S.H., Höner zu Siederdissen,C., Tafer,H., Flamm,C., Stadler,P.F. and Hofacker,I.L. (2011) ViennaRNA Package 2.0. *Algorithms Mol. Biol.*, **6**, 26.

3. He,B., Rong,M., Lyakhov,D., Gartenstein,H., Diaz,G., Castagna,R., McAllister,W.T. and Durbin,R.K. (1997) Rapid Mutagenesis and Purification of Phage RNA Polymerases. *Protein Expr. Purif.*, **9**, 142–151.

4. Filonov,G.S., Kam,C.W., Song,W. and Jaffrey,S.R. (2015) In-Gel Imaging of RNA Processing Using Broccoli Reveals Optimal Aptamer Expression Strategies. *Chem. Biol.*, **22**, 649–660.

5. Masek,T., Vopalensky,V., Suchomelova,P. and Pospisek,M. (2005) Denaturing RNA electrophoresis in TAE agarose gels. *Anal. Biochem.*, **336**, 46–50.

6. Kibbe,W.A. (2007) OligoCalc: an online oligonucleotide properties calculator. *Nucleic Acids Res.*, **35**, W43–W46.
